# Genomic testing in 1019 individuals from 349 Pakistani families results in high diagnostic yield and clinical utility

**DOI:** 10.1038/s41525-020-00150-z

**Published:** 2020-10-05

**Authors:** Huma Cheema, Aida M. Bertoli-Avella, Volha Skrahina, Muhammad Nadeem Anjum, Nadia Waheed, Anjum Saeed, Christian Beetz, Jordi Perez-Lopez, Maria Eugenia Rocha, Salem Alawbathani, Catarina Pereira, Marina Hovakimyan, Irene Rosita Pia Patric, Omid Paknia, Najim Ameziane, Claudia Cozma, Peter Bauer, Arndt Rolfs

**Affiliations:** 1 Pediatric Department of Gastroenterology, Children’s Hospital of Lahore Hospital, Lahore, Pakistan; 2 CENTOGENE AG, Rostock, Germany; 3 University of Rostock, Rostock, Germany

**Keywords:** Molecular medicine, Genetics research, Medical genetics, Molecular medicine, Genetics research

## Abstract

We implemented a collaborative diagnostic program in Lahore (Pakistan) aiming to establish the genetic diagnosis, and to asses diagnostic yield and clinical impact in patients with suspected genetic diseases. Local physicians ascertained pediatric patients who had no previous access to genetic testing. More than 1586 genetic tests were performed in 1019 individuals (349 index cases, 670 relatives). Most frequently performed tests were exome/genome sequencing (ES/GS, 284/78 index cases) and specific gene panels (55 index cases). In 61.3% of the patients (*n* = 214) a genetic diagnosis was established based on pathogenic and likely pathogenic variants. Diagnostic yield was higher in consanguineous families (60.1 vs. 39.5%). In 27 patients, genetic diagnosis relied on additional biochemical testing, allowing rapid assessment of the functional effect of the variants. Remarkably, the genetic diagnosis had a direct impact on clinical management. Most relevant consequences were therapy related such as initiation of the appropriated treatment in a timely manner in 51.9% of the patients (*n* = 111). Finally, we report 12 candidate genes among 66 cases with no genetic diagnosis. Importantly, three of these genes were validated as ‘diagnostic’ genes given the strong evidence supporting causality derived from our data repository *(CAP2-*dilated cardiomyopathy*, ITFG2-*intellectual disability and *USP53-*liver cholestasis). The high diagnostic yield, clinical impact, and research findings demonstrate the utility of genomic testing, especially when used as first-line genetic test. For patients with suspected genetic diseases from resource-limited regions, ES can be considered as the test of choice to achieve genetic diagnosis.

## Introduction

Genetic diseases are often severely disabling and have detrimental impact on patients’ physical and cognitive abilities. Such lifelong impairments also have a considerable impact on affected families^1^. Genetic testing aimed at establishing a precise diagnosis in patients with genetic diseases is extremely important in order to alleviate the disease burden in these families. An accurate diagnosis will guide clinical decisions, allowing for personalized medical management, monitoring and more accurate prognosis. It is of maximum relevance in cases with treatable genetic diseases, where establishing a specific treatment program can make a major difference in outcome^2–5^. For the families, establishing a diagnosis allows for genetic counseling, including information on recurrence risk, and facilitates family planning and reproductive choices. An accurate diagnosis also advances access to information and assistance from patient support groups as well as access to the education, health, and social care systems, ultimately forming the basis for research into new therapies^6^.

Unfortunately, access to genetic services and genetic testing is limited in developing countries^7^. Recently introduced diagnostic technologies, such as exome and genome sequencing (ES/GS), which have a high diagnostic and clinical utility^8^, are limited for patients in these countries. We implemented a collaborative diagnostic program in Lahore, Pakistan. Diagnostic testing was offered to patients suffering from (suspected) genetic diseases who have limited or no access to genetic testing due to economical or geographical reasons. Through this collaboration program, local clinicians selected patients based on clinical presentation and suspicion of an underlying genetic disease. For 270 index patients (77.4%) ES was performed as the first-line genetic test. GS was indicated as reflex testing in complex cases where ES testing produced no diagnosis.

Here we report the results from 1586 genetic tests performed on 1019 individuals, aimed at establishing the genetic diagnoses. The high diagnostic yield, clinical utility, and relevant research findings demonstrate the usefulness of genomic testing, especially when applied as first-line genetic test.

## Results

A total of 245 patients presented with a clinical phenotype at a very young age, having an early disease onset (from prenatal to 5 years old). Approximately half of the patients had a positive family history suggesting a genetic etiology (*n* = 179). In addition, 295 families reported parental consanguinity, which was expected given the geographical origin of the patients, where intra-familiar marriages are more commonplace. The demographics of all 349 index cases are summarized in Table 1.

**Table 1:** Demographics of the complete cohort (349 patients) and cases with genetic diagnosis (P/LP variants identified), uncertain (VUS) and no diagnoses (no relevant variant identified).

Features	All index cases *n* = 349	Patients with genetic diagnosis *n* = 214	Patients with uncertain diagnosis *n* = 69	Patients with no diagnosis *n* = 66	Chi-square *p* value
		(% relative to total number of cases in the category)	(% relative to total number of cases in the category)	(% relative to total number of cases in the category)	
Age at onset					
Prenatal	45	29 (64.4%)	10 (22.2%)	6 (13.3%)	0.352
0–5 years old	200	119 (59.5%)	43 (21.5%)	38 (19.0%)	
>5 years old	33	21 (63.6%)	3 (9.1%)	9 (27.3%)	
Not provided	71	45	13	13	
Family history					
Positive	179	109 (60.9%)	38 (21.2%)	32 (17.9%)	0.886
Negative	150	92 (61.4%)	29 (19.3%)	29 (19.3%)	
Unknown	20	13	2	5	
Consanguinity					
Yes	295	186 (60.1%)	57 (19.3%)	52 (17.6%)	0.02
No	38	15 (39.5%)	12 (31.6%)	11 (28.9%)	
Unknown	16	13	0	3	

As early disease onset, positive family history, and parental consanguinity can indicate a genetic origin of the disease, we evaluated if any of these factors influenced diagnostic yield (Table 1). Indeed, diagnostic yield was higher in consanguineous families, while family history and age at disease onset had no effect (Table 1).

### Motive of referral

To assess clinical presentation and motive of referral, we analyzed clinical information based on HPO terms. The top reported disease categories were abnormality of the metabolism and abnormality of the digestive system. Similarly, most reported HPOs were hepatomegaly (69 index cases), splenomegaly (39 cases), elevated hepatic transaminase (34 cases), abdominal distention (31 cases), and jaundice (24 cases). This is corresponding with the main referral clinical department (Gastroenterology). The second most reported disease category was abnormality of the nervous system, with motor delay (51 cases), delayed speech and language development (38 cases), developmental regression (36 cases), and global developmental delay (30 cases) being the top reported HPOs. A summary of the reported phenotypes and corresponding HPOs are depicted in Supplementary Fig. 1.

### Diagnostic yield

For ES, trio testing was performed (parents and affected child). In 15 families, biological material from deceased children with suspected genetic diseases was not available. Genetic testing was performed on the parents, aiming to identify variants that could explain the phenotype of their deceased children.

ES was the most frequently chosen method. For 270 patients (77.4%), ES was selected as the first-line test, followed by GS in 78 cases. A summary of the testing strategy in 349 index cases is presented in Supplementary Fig. 2.

**Fig. 1:**
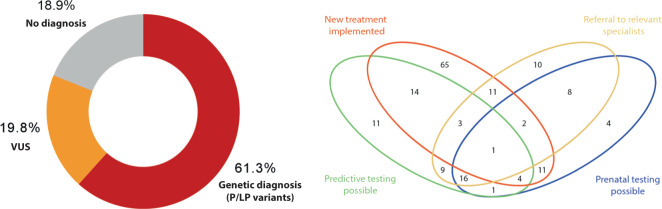
High diagnostic yield and clinical utility. **a** High diagnostic yield obtained in 349 index patients. In 214 patients (61.3%) a genetic diagnosis was established based on pathogenic/likely pathogenic variants. **b** High clinical impact of genetic testing with 170 patients (79.4%) reported with a change in clinical management after establishment of the genetic diagnosis. Venn diagram representing the main categories evaluated and the number of index cases per category/combination.

Pathogenic and likely pathogenic variants were identified in 61.3% of the patients (*n* = 214), establishing a genetic diagnosis. In addition, in 19.8% (69 patients), VUS were identified (Fig. 1a). The majority of the VUS were formally classified as such, but with strong evidence supporting pathogenicity and clinical interpretation consistent with a ‘potential’ genetic diagnosis (46 patients).

The most frequently detected type of variants were single nucleotide variants (SNVs), with 191 P/LP variants reported. These included recurrent disease-causing variants for glycogen storage disease IIIa/IIIb (*AGL*), progressive familial intrahepatic cholestasis type 1 (*ATP8B1*), and Niemann–Pick disease type A/B (*SMPD1*). In addition, 20 P/LP copy number variants (CNVs) were reported. Two recurrent CNVs were detected, the *SMN1* deletion of exon 7–8 and a duplication within *PRSS1* (detected in three cases each). CNV detection was the main reason leading to GS-based diagnosis after inconclusive ES (mainly involving 1–2 exons). CNVs including large chromosomal areas were detected in three patients with Turner syndrome, partial chromosome 22q11.2 deletion syndrome, and partial trisomy 11q. All 179 unique P/LP variants are listed in Supplementary Table 1.

We also explored the mode of inheritance of diseases identified in patients with consanguineous parents, compared to patients without or unknown consanguinity. As expected, a larger proportion of autosomal recessive diseases were diagnosed in the consanguineous group (88.7 vs. 57.1%). They presented less autosomal dominant (AD) and X-linked (XL) diseases compared to the rest of the cases (8.6% AD–1.6% XL vs. 28.6% AD–10.7% XL).

### Main diagnosed diseases

Corresponding with main motive of referral, 100 patients (46.7%) were diagnosed with genetic metabolic diseases, including glycogen storage disease, Niemann–Pick disease, biotinidase deficiency, mucopolysaccharidosis, and tyrosinemia. Fifty-two cases (24.4%) were diagnosed with genetic disease of the digestive system, such as early onset hereditary pancreatitis and familial intrahepatic cholestasis. In 41 patients (19.2%), multisystem genetic diseases were diagnosed, including Kleefstra syndrome, Noonan syndrome, Hennekam syndrome, Cantu syndrome, and Raynaud-Claes syndrome. Neurological diseases were detected in 18 patients (8.4%) (e.g., early infantile epileptic encephalopathy, spastic paraplegia). Three patients presented deletions/duplication involving a large chromosomal region (1.4%). A list of all diagnosed diseases is presented in Supplementary Table 2.

**Fig. 2:**
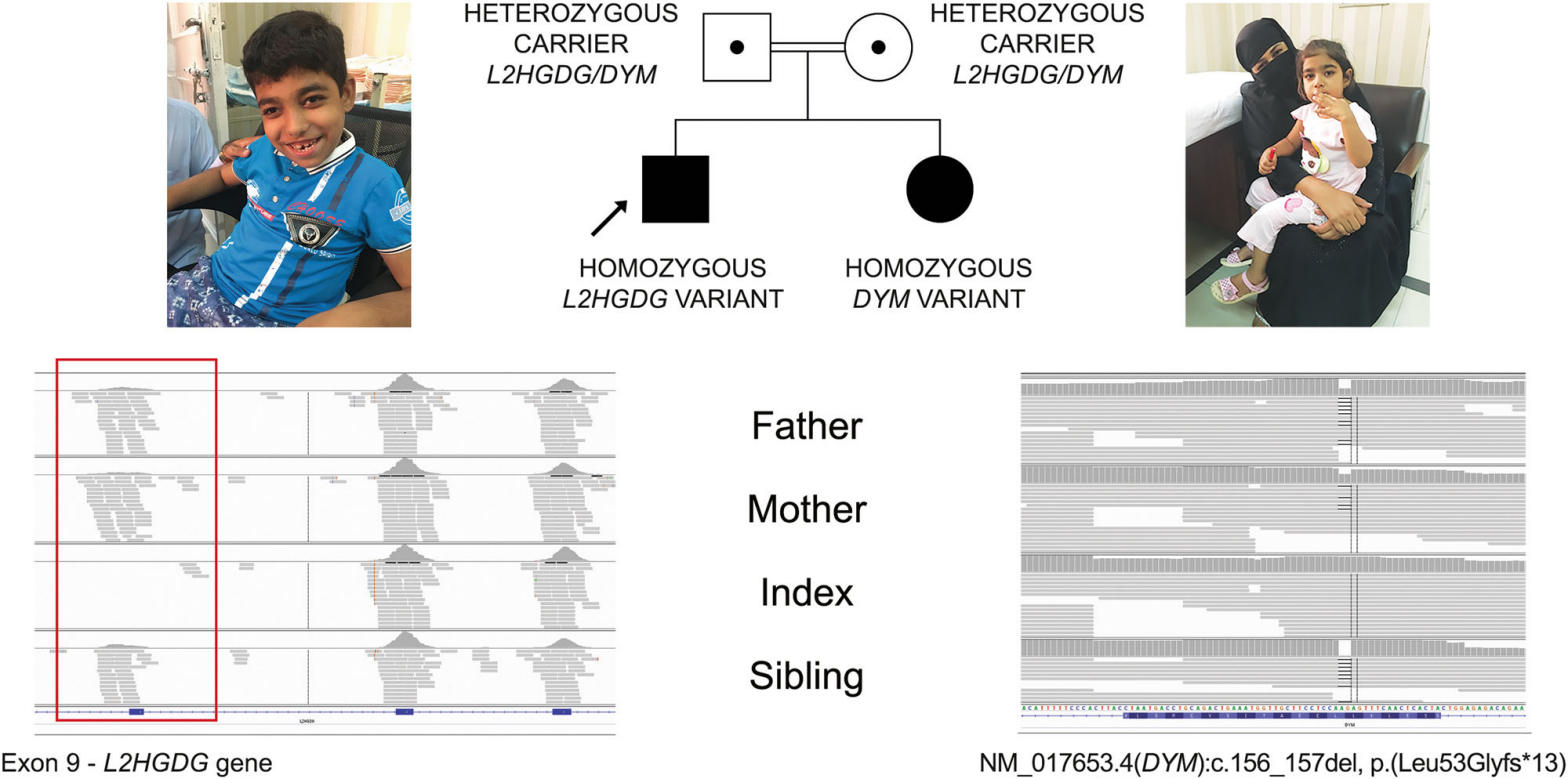
Two distinct genetic diseases in one family. *Left:* Upper body photograph of male index patient at age of 10 years, with mild dysmorphic features: narrow forehead, thick eyebrows, long eyelashes, mildly upslanted eyes, short palpebral fissure, broad nasal bridge, low hanging columella, short philtrum, thin upper lip and wide mouth, dental malalignment with delayed eruption. A homozygous pathogenic deletion of exon 9 in the *L2HGDH* gene was detected in this patient by ES. IGV image showing the corresponding region of *L2HGDH*, with the absence of reads in index patient (area corresponding to exon 9 in red box). Parents and siblings have reduced number of reads consistent with a heterozygous deletion (exon 8 and 10: 100–195 reads, exon 9: 23–35 reads). The findings were confirmed by qPCR. *Right:* Photograph of female sibling at age of 5 years. A homozygous likely pathogenic variant in the *DYM* gene was detected in the affected sibling (variant quality score (QS) = 1072, reference value >215^38^). Carrier status of both parents was confirmed. The variant was validated by Sanger sequencing. Parents of the patients provided written informed consent for the use of patient images for scientific publication.

An interesting family with the diagnosis of two distinct genetic diseases is presented in Fig. 2. Parents were consanguineous, with two affected children. Clinical presentations indicated divergent phenotypes in the siblings. The male index had neurodevelopmental delay, seizures, ataxia, and leukodystrophy. The female sibling presented neurodevelopmental delay, regression, and microcephaly. Using ES, we identified a homozygous pathogenic deletion encompassing exon 9 of the *L2HGDH* in the index, and a homozygous LP frameshift variant in *DYM* (NM_017653.4:c.156_157del, p.(Leu53Glyfs*13)) in the affected sibling. These findings allowed diagnoses of L-2-hydroxyglutaric aciduria in the index and Dyggve–Melchior–Clausen disease in the affected sister to be established. Parents were confirmed as heterozygous carriers of both variants (Fig. 2).

Furthermore, dual genetic diagnoses were made in 2.1% of the index patients (6/285 index cases with ES/GS). Clinical and genetic details of these patients are summarized in Supplementary Table 3. In four of these cases, one of the diagnosis relates to genetic disorders that are relatively frequent in the studied population (chronic pancreatitis and G6PD deficiency).

### Strength of clinical, genetic, and biochemical data combination

In 27 patients, the genetic diagnosis was established using a combination of clinical, genetic, and biochemical data (Table 2). Biochemical testing allowed for supporting evidence for variant pathogenicity, classification, and diagnosis. This was especially relevant for nine patients with novel or very rare variants that otherwise would be classified as VUS due to the lack of functional evidence (*MAN2B1, NAGLU, NPC1, SMPD1, GLB1* genes). In other cases, parallel biochemical testing confirmed variant classification based on previous publications or upgraded the classification from LP to P. Most frequently diagnosed diseases were Niemann–Pick disease type A/B and type C1, Mucopolysaccharidosis type IIIB and type IVA, and GM1-gangliosidosis type I (Table 2).

**Table 2:** Homozygous variants detected in 27 patients with subsequent abnormal biochemical results.

Gene	DNA change^a^	Protein change	Disease (OMIM®)	Biochemical test result	PMID	ACMG criteria
*MAN2B1*	NC_000019.9(NM_000528.3):c.1644+4A>G	Splicing	Alpha-Mannosidosis (248500)	Alpha mannosidase < 1.9 (LOD) µmol/l/h	Very rare, first time as homozygous	PS3*, PM2, PP3, PP4
*GBA*	NM_000157.3:c.1448T>C	p.(Leu483Pro)	Gaucher disease, type I (230800)	Beta-glucocerebrosidase < 1 (LOQ) μmol/L/h; lyso-Gb1 = 401 ng/mL	PMIDs 2880291, 8294487, 8160756	PS1, PS3, PM2, PP3, PP4
*NAGLU*	NM_000263.3:c.1336G>A	p.(Glu446Lys)	Mucopolysaccharidosis type IIIB (252920)	Alpha-N-acetylglucosaminidase < 0.3 (LOQ) μmol/L/h	PMID 14984474	PS1, PS3, PM2, PP3, PP4
*NAGLU*	NM_000263.3:c.291T>G	p.(Cys97Trp)	Mucopolysaccharidosis type IIIB (252920)	Alpha-N-acetylglucosaminidase < 0.3 (LOQ) μmol/L/h	Very rare, first time as homozygous	PS3*, PM2, PP3, PP4
*NAGLU*	NM_000263.3:c.701G>C	p.(Arg234Pro)	Mucopolysaccharidosistype IIIB (252920)	Alpha-N-acetylglucosaminidase < 0.3 (LOQ) μmol/L/h	PMID 9832037	PS1, PS3, PM2, PP3, PP4
*NPC1*	NM_000271.4:c.1097C>G	p.(Ser366*)	Niemann-Pick disease type C1 (257220)	Lyso-SM-509 = 1.7 ng/mL; lyso-SM-465 = 14.5 ng/mL	Novel variant	PVS1, PS3, PM2
*NPC1*	NM_000271.4:c.2608T>A	p.Ser870Thr	Niemann-Pick disease type C1 (257220)	Lyso-SM-509 = 2.4 ng/mL; lyso-SM-465 = 22.9 ng/mL	Novel variant	PS3*, PM2, PP3
*NPC1*	NM_000271.4:c.2978dup	p.(Asp994Argfs*13)	Niemann-Pick disease type C1 (257220)	Lyso-SM-509 = 3.8 ng/mL; lyso-SM-465 = 17.1 ng/mL	Very rare, first time as homozygous	PVS1, PS3, PM2
*NPC1*	NM_000271.4:c.3020C>T	p.(Pro1007Leu)	Niemann-Pick disease type C1 (257220)	Lyso-SM-509 = 2.7 ng/mL; lyso-SM-465 = 24.3 ng/mL	PMIDs 17160617, 26666848	PS1, PS3, PM2, PM3, PM5, PP3
*NPC1*	NM_000271.4:c.3503G>A	p.(Cys1168Tyr)	Niemann-Pick disease type C1 (257220)	Lyso-SM-509 = 3.2 ng/mL; lyso-SM-465 = 30 ng/mL	PMID 11333381	PS3*, PM2, PP3, PP5
*HEXA*	NM_000520.5:c.109T>A	p.(Tyr37Asn)	Tay-Sachs disease (272800)	Hexosaminidase A < 0.09 (LOD) µmol/L/h; total hexosaminidase = 13.7 μmol/L/h	PMID 18358410	PS3*, PM2, PM3, PP5
*HEXA*	NM_000520.5:c.902T>G	p.(Met301Arg)	Tay-Sachs disease (272800)	Hexosaminidase A < 0.09 (LOD) μmol/L/; total hexosaminidase = 7.3 μmol/L/h	PMIDs 8044648, 18490185	PS3*, PM2, PP3, PP4, PP5
*HEXA*	NM_000520.4:c.1274_1277dup	p.(Tyr427Ilefs*5)	Tay-Sachs disease (272800)	Hexosaminidase A < 0.09 (LOD) μmol/L/h; total hexosaminidase = 16.2 μmol/L/h	PMIDs 2848800, 21228398, 22975760	PVS1, PS3, PM2, PP5
*HEXB*	NM_000521.3:c.1597C>T	p.(Arg533Cys)	Sandhoff disease (268800)	Hexosaminidase A = 0.5 μmol/L/h; total hexosaminidase = 0.6 μmol/L/h	PMIDs 21567908, 22848519	PS3, PM2, PM3, PM5, PP3, PP5
*HEXB*	NM_000521.3:c.850C>T	p.(Arg284*)	Sandhoff disease (268800)	Beta-hexosaminidase A = 0.4 μmol/L/h; total hexosaminidase = 0.8 μmol/L/h	PMIDs 8162015, 24613245, 25525159	PVS1, PS3, PM2, PM3, PP5
*SMPD1*	NM_000543.4:c.1382_1383del	p.(His461Argfs*3)	Niemann-Pick disease type A/B (257200/607616)	Acidic sphingomyelinase < 0.4 (LOD) μmol/l/h, lyso-SM-509 = 6.3 ng/mL; lyso-SM-465 = 747 ng/mL	Very rare, homozygote patient in CentoMD® with increased biomarker	PVS1, PS3, PM2, PP4
*SMPD1*	NM_000543.4:c.1493G>A	p.(Arg498His)	Niemann-Pick disease type A/B (257200/607616)	Acidic sphingomyelinase < 0.4 (LOD) μmol/L/h; lyso-SM-509 = 2.9 ng/mL, lyso-SM-465 = 534 ng/mL	PMID 15221801	PS1, PS3, PM2, PP3, PP4
*SMPD1*	NM_000543.4:c.1624C>T	p.(Arg542*)	Niemann-Pick disease type A/B (257200/607616)	Acidic sphingomyelinase < 0.4 (LOD) μmol/L/h; lyso-SM-509 = 5.5 ng/mL; lyso-SM-465 = 1480 ng/mL	PMIDs 22796693, 23188845	PVS1, PS3, PM2, PM3, PP5
*SMPD1*	NM_000543.4:c.1624C>T	p.(Arg542*)	Niemann-Pick disease type A/B (257200/607616)	Acidic sphingomyelinase < 0.4 (LOD) μmol/L/h; lyso-SM-509 = 6.3 ng/mL; lyso-SM-465 = 675 ng/mL	PMIDs 22796693, 23188845	PVS1, PS3, PM2, PM3, PP5
*SMPD1*	NM_000543.4:c.1624C>T	p.(Arg542*)	Niemann-Pick disease type A/B (257200/607616)	Acidic sphingomyelinase < 0.4 (LOD) μmol/L/h; lyso-SM-509 = 5.4 ng/mL; lyso-SM-465 = 870 ng/mL	PMIDs 22796693, 23188845	PVS1, PS3, PM2, PM3, PP5
*SMPD1*	NM_000543.4:c.314T>C	p.(Leu105Pro)	Niemann-Pick disease type A/B (257200/607616)	Acidic sphingomyelinase 3.9 μmol/L/h; lyso-SM-509 = 3.2 ng/mL; lyso-SM-465 = 401 ng/mL	PMIDs 15221801, 16010684	PS1, PS3, PM2, PP3
*SMPD1*	NM_000543.4:c.748A>C	p. (Ser250Arg)	Niemann-Pick disease type A/B (257200/607616)	Acidic sphingomyelinase < 0.4 (LOD) μmol/L/h; lyso-SM-509 = 10.4 ng/mL; lyso-SM-465 = 885 ng/mL	PMIDs 12556236	PS3, PM2, PM3, PP1, PP3, PP5
*GLB1*	NM_001317040.1:c.1025_1026delAT	p.Tyr342fs	GM1-gangliosidosis type I (230500)	Beta-galactosidase < 3.3 (LOQ) μmol/L/h	Very rare, 2 unrelated homozygous patients in CentoMD®	PVS1, PS3, PM2
*GLB1*	NM_001317040.1:c.1398C>G	p.Tyr466*	GM1-gangliosidosis type I (230500)	Beta-galactosidase = 4.1 µmol/L/h	Novel variant	PVS1, PS3, PM2
*GLB1*	NM_001317040.1:c.1399C>T	p.(Arg467Trp)	GM1-gangliosidosis type I (230500)	Beta-galactosidase < 1 (LOD) μmol/L/h	Very rare, first time as homozygous	PS3*, PM2, PP3, PP4
*GALNS*	NM_001323544.1:c.470C>T	p.(Pro157Leu)	Mucopolysaccharidosis type IVA (253000)	Galactosamine-6-sulfate sulfatase < 0.1 (LOD) μmol/L/h	PMIDs 7633425, 8651279, 22940367	PS3, PM2, PM5, PP3, PP4
*GALNS*	NM_001323544.1:c.516C>G	p.(His172Gln)	Mucopolysaccharidosis type IVA (253000)	Galactosamine-6- sulfate sulfatase = 1.7 µmol/L/h	PMIDs 9375852, 22940367	PS3, PM2, PM5, PP3, PP4

PS3* Classification raised from LP to P given the clearly pathological biochemical result.

Reference values: alpha mannosidase ≥ 16.2 µmol/L/h, lyso-SM-509 ≤ 0,9 ng/mL, beta-glucocerebrosidase ≥ 4.1 μmol/L/h; lyso-Gb1 ≤ 6,8 ng/mL; alpha-N-acetylglucosaminidase ≥ 1.5 μmol/L/h; lyso-SM-465 = 46.3 ng/mL; hexosaminidase A ≥ 2.0 μmol/L/h; total hexosaminidase ≥ 4.5 μmol/L/h; acidic sphingomyelinase ≥ 1.7 μmol/L/h; beta-galactosidase ≥ 28.5 μmol/L/h; galactosamine-6-sulfate sulfatase ≥ 2.0 μmol/L/h.

All variants were classified as pathogenic based on abnormal enzyme and/or biomarker results, previous publication(s), and/or additional patients in our data repository.

*LOD* lower limit of detection, *LOQ* lower limit of quantification.

^a^Nomenclature of DNA variants according to HGVS recommendations, including intronic variants (NG_008318.1(NM_000528.3):c.1644+4A>G). All variants were checked with Mutalyzer to ensure correct nomenclature.

### Clinical utility

Aiming to evaluate clinical utility of the genetic testing, referring physicians reported the main changes on clinical management of the patients and families that had received a genetic diagnosis. For all patients (*n* = 214) changes in general management were reported (e.g. modification in life style, avoidance of decompensating agents, initiatiation of special surveillance). In 79.4% of the patients (*n* = 170) more specific measures were reported. Therapy related changes, such as initiation of the appropriated treatment were reported in 51.9% (*n* = 111). Relevant examples included: initiating treatment with nitisinone in a patient with tyrosinemia type I and bone marrow transplantation in several patients with immunological diseases (immunodeficiencies, lymphoproliferative syndrome, agammaglobulinemia). Furthermore, referral to other relevant medical specialties was reported in 28.0% of the patients (*n* = 60). Establishment of the genetic diagnosis guided further screening/surveillance of other affected or at-risk individuals (27.6%, *n* = 59) or made prenatal testing possible in following pregnancies (22.0%, *n* = 47). Detailed results are shown in Fig. 1b and Supplementary Table 2.

### Research findings in cases with no genetic diagnosis and validation of ‘research’ genes

A total of 337 families consented to extensive evaluation of the genetic data beyond the known ‘diagnostic’ genes with established genotype–phenotype relationship. Aiming to identify new candidate genes, we performed further analysis of the sequencing data in 66 patients with no diagnosis after ES/GS testing. Remarkably, in 12 cases (18.2% of the patients with no diagnosis), we identified variants in candidate genes, illustrating the importance of this cohort for research and new gene discovery.

All relevant research findings and the respective patient’s phenotype are summarized in Supplementary Table 4. Selected variants were novel or very rare, mainly homozygous, and with high predicted impact on protein function (e.g., nonsense, frameshift, splicing). Co-segregation in the family (e.g., similarly affected siblings), published evidence on gene function, animal models, or previous case reports were considered. Next, we analyzed phenotype–genotype data from our repository, searching for additional cases supporting the causal role of the variants in the respective genes. Twelve candidate genes were identified for several early onset diseases (Supplementary Table 4). Importantly, three of these candidate genes were validated and confirmed as ‘diagnostics’ considering previous publications and our newly identified patients *(ITFG2, USP53*, and *CAP2)*.

**Fig. 3:**
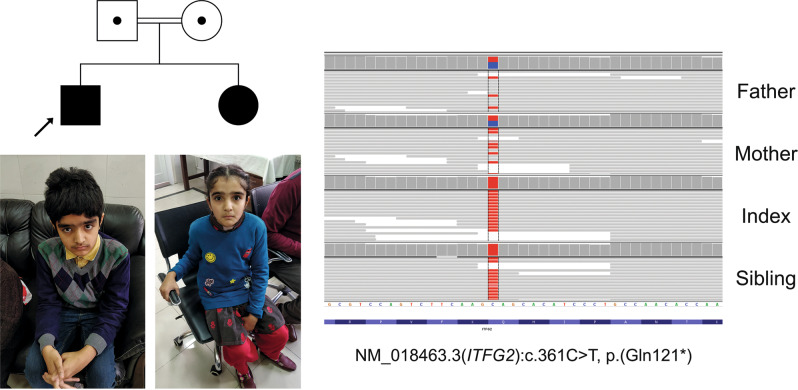
*ITFG2* is a newly identified gene related to neurodevelopmental delay and ataxia. Index and sister are homozygous for NM_018463.3:c.361C>T, p.Gln121* and parents are confirmed heterozygous carriers. Photographs of male index (12 years old) and female sibling (10 years old) showing mild dysmorphic features. Male index: thick hair, narrow forehead, bushy eyebrows with synophris, almond-shape eyes, long eyelashes, broad nasal bridge, short columella, short and marked philtrum with Cupid bow and small mouth. Sister: narrow forehead, bushy eyebrows with synophris, small eyes almond shaped, short palpebral fissure, long eyelashes, broad and tall nasal bridge, thin lips with small mouth. Corresponding IGV image in exon 4 of *ITFG2* is shown (variant QS = 5165 and 4733 in index and sibling, respectively). Parents of the patients provided written informed consent for the use of their children images in scientific publication.

One relevant example relates to the *ITFG2* gene. The male index of consanguineous parents, presented with NDD, seizures, developmental regression, and ataxia. A female sibling was similarly affected. Despite clear suspicion of a genetic disease, no relevant variant was identified in known ‘diagnostic’ genes. Additional evaluation focusing on regions of homozygosity shared by the patients identified a homozygous nonsense variant in *ITFG2* (Fig. 3). The gene was considered the top candidate based on a previous report of a homozygous nonsense variant in patients with intellectual disability from an unrelated consanguineous family^9^. Subsequent analysis of our data repository identified three additional unrelated patients with overlapping phenotypes, including NDD and ataxia, with homozygous loss of function variants in *ITFG2* (NM_018463.3:c.848-1G>A; NM_018463.3:c.704dupC, p.(Ala236fs), NM_018463.3:c.1000_1001delAT, p.(Ile334fs)). This finding established the genetic diagnosis, producing immediate benefits for all four families.

A similar example refers to a form of early onset cholestasis with hepatomegaly (*USP53* gene). The male index presented at 8 months old with jaundice, itching, and pigmented stools. Elevated liver transaminases and bilirubin were detected in blood, and intrahepatic cholestasis was seen in a liver biopsy. Family history was positive for a similarly affected male sibling, suggesting an autosomal recessive or XL disease. A novel homozygous nonsense variant was detected in *USP53*, a gene not associated with any phenotype in OMIM (last accessed 8 January 2020). Recently, Maddirevula et al. reported the gene as a candidate for cholestatic liver disease based on a consanguineous family with two children having a homozygous frameshift variant in *USP53*^10^. In addition, five unrelated patients were identified in our data repository. These patients presented a similar phenotype, mainly including early onset intrahepatic cholestasis and presented five distinct homozygous likely LoF variants in *USP53* NM_019050.2:c.169C>T, (p.Arg57*); NM_019050.2:c.475_476delCT, (p.Leu159fs); NM_019050.2:c.822+1delG, p:?; NM_019050.2: c.951delT, (p.Phe317fs); NM_019050.2:c.1214dupA, (p.Asn405fs). These results strongly indicate that biallelic loss of function variants in *USP53* are causal for an autosomal recessive form of early onset cholestatic liver disease.

The third gene relates to a severe early onset form of dilated cardiomyopathy (DCM) and congenital heart defects (*CAP2* gene). Family history was positive, with two deceased siblings with similar phenotype. A nonsense, homozygous variant was detected in *CAP2*. The gene was considered as a candidate because of the phenotype observed in Cap2-null mice with cardiomyopathy and cardiac conduction disorder^11^. Furthermore, Aspit et al. recently reported two children with DCM from a consanguineous Beduine family. Both patients had a homozygous variant affecting the donor splice site of *CAP2* exon 7, which causes skipping of exons 6 and 7 in patient-derived fibroblasts^12^. Taking both studies into account, these findings strongly suggest a role of *CAP2* in DCM and the importance of this gene for normal function of the human heart.

All referring clinicians were re-contacted to notify the new diagnoses established based on our data repository.

### Secondary findings

Secondary findings were evaluated for the 59 actionable genes according to the latest recommendations of the ACMG^13^. A total of 337 families in this study were interested to know about secondary findings, and therefore provided signed informed consent. Only 12 families did not wish to be informed about such findings. Four heterozygous P/LP variants were reported in these actionable genes, namely *MYBPC3, MYH6, KCNQ1*, and *BRCA1*.

## Discussion

In this cohort, we have shown the excellent diagnostic value of ES/GS as first-line testing in patients with suspected genetic diseases. In 61.3% of the patients, a genetic diagnosis was established (Fig. 1a).

Similar reports applying ES/GS as first-line testing in developing countries to achieve genetic diagnosis are scarce. Two recent studies describe their results after performing ES/GS in patients with limited or no previous access to genetic testing. The first study was developed in China and included 1323 patients for whom a ‘mini’ clinical exome was performed in a proband-only-based design. The ES included only 2742 diagnostic genes^14^. Hu et al. reported a diagnostic yield of 28.8%, which is considerable, but well below the 61.3% yield detected in our cohort. The limited number of assessed genes, singleton testing, and lack of genetic training among the doctors referring the patients may explain the lower diagnostic yield of the Chinese study^14^.

A second study included a smaller number of patients (60 cases) with suspected genetic diseases from Mexico^15^. Scocchia et al. reported a high diagnostic yield (68.3%) after GS was used as first-line diagnostic testing. Almost half of the patients presented relatively large CNVs (microdeletions), which could be expected based on the clinical selection with 76.7% of the patients presenting a phenotype consistent with malformation patterns. Notably, the genetic diagnosis influenced clinical management in 48.8% (*n* = 20) of the cases.

High diagnostic and clinical utility of ES/GS when used as first-line diagnostic test has been demonstrated also in studies from developed countries. French et al. and Stark et al. (United Kingdom and Australia) showed a diagnostic yield of 57.5% (80 patients with ES) and 21% (195 patients with GS), respectively. Similarly, both studies reported a relevant clinical utility (32.6% and up to 48%), concluding that ES/GS are valuable as first-line diagnostic tests^16,17^.

Several factors are known to influence ES/GS diagnostic yield. Sample size of the reported cohort, consanguinity, and design of testing (trio vs. singleton) are reported to significantly influence diagnostic yield^8^. The used technology (ES vs. GS), the training of genetic scientists evaluating the data, and establishing genotype–phenotype connections are other recognized factors directly related to the testing/analysis procedure. Finally, a relevant factor is the accuracy of the patients’ clinical evaluation as well as selection and precise communication of the clinical phenotype to the laboratory specialists evaluating the genetic data.

In this cohort of 349 index patients, diagnostic yield was higher in consanguineous families (60.1 vs. 39.5%). Similarly, Alfares et al., reported a diagnostic yield of 49% in patients with a wide range of clinical presentations (222/454). Diagnostic yield was 53% among consanguineous families vs. 39% in patients from non-consanguineous marriages^18^. Al-Dewik et al., established the genetic diagnosis in 48.3% patients with rare genetic disorders (246/509). Diagnostic yield was higher in consanguineous families (52.4 vs. 39.5%)^19^. Al-Shamsi et al. reported 50% ES diagnostic yield among 85 patients suspected to have inborn errors of metabolism^20^. All patients originated from the Middle Eastern region. These studies consistently reported high diagnostic yield among consanguineous families, yet, below the yield reported here.

We consider that the testing technology in combination with the detailed clinical assessment and the analysis of the family history, positively influenced the high diagnostic yield in this study. In addition, the combination of clinical, genetic, and biochemical data facilitated genetic diagnosis in many cases, especially in this cohort greatly composed by patients with metabolic diseases. Novel or very rare variants, which would otherwise be classified as VUS, were classified as pathogenic given the clear pathological biochemical results (enzyme and/or biomarker).

Sending biological samples for genetic testing across country borders is a complex procedure; complicated packaging is necessary to ensure safe handling of the samples during transport, with increased shipping costs. In this study, we have used filter cards (CentoCard®) for handling and transportation of biological material (dried blood spots, DBS) from Pakistan to Germany. The use of DBS makes genetic, enzymatic, and biomarker testing possible. This efficient system of sample collection and transportation can be easily applied in similar studies and it is even more relevant in times of pandemic outbreaks.

A known advantage of ES/GS is the possibility of unbiased assessment of genes. As a result, dual diagnoses can be established. These patients usually present with a wide clinical spectrum representing blended phenotypes. In this study, we identified dual diagnoses in 2.1% of the cases. Similarly, Monies et al., reported dual molecular diagnoses in 1.5% of cases from a highly consanguineous population^21^. We previously reported dual diagnoses in 1% of 1000 ES cases^22^. Dual molecular diagnoses were detected in 1.2% (148/11,877) according to the analysis performed by Balci et al., which included five different studies^23^.

Besides differences in study designs from Schoccia et al. and Hu et al., both groups reported high impact of test results on clinical management (48.8% and 45.1%, respectively), such as referral to other specialties for complementary care, avoiding further diagnostic interventions (e.g., biopsy), starting or discontinuing a therapy, and modifying (post-test) genetic counseling^14,15^.

In our study the genetic diagnosis had a larger impact on clinical management. Especially relevant were therapy related decisions, such as initiation of the appropriate treatment in a timely manner implemented in 51.9% of the patients. Clinical utility is even more important in developing countries with limited resources and access to health care. Having a specific diagnosis allows careful planning of the available resources. As previously reported, the use of ES as first-line testing is cost-effective, as compared with the standard diagnostic (stepwise) pathway^24^. Application of ES results in a considerable reduction of health care costs, even more so when applied earlier in the diagnostic trajectory^25^.

Results from this study could serve as the foundation for implementation of national or regional programs aiming to reduce the burden of severe genetic conditions, and to facilitate the medical care and timely implementation of appropriated therapies for these patients. We show that genomic testing is a valuable diagnostic strategy in developing countries with limited access to genetic testing, especially in populations with elevated consanguinity rate.

In conclusion, our results indicate the excellent diagnostic and clinical value of a genomic approach as first-line diagnostic testing when combined with careful clinical evaluation and patient selection. Specifically, for patients with suspected genetic diseases from resource-limited regions, ES can be considered as the test of choice to achieve genetic diagnosis. In this cohort, ES/GS allowed for genetic research and validation of new gene-phenotype associations, giving hope to previously undiagnosed patients.

## Methods

### Patients

Patients were selected by local physicians from the Children’s Hospital of Lahore (Pakistan) based on careful evaluation of the phenotype and strong suspicion of an underlying genetic disorder. The Children’s Hospital of Lahore is a tertiary referral center receiving patients from the Punjab province and the rest of the country. This diverse population comprises at least 8–9 different ethnic groups. Most patients were referred from the Gastroenterology department, other referrals included cases from Neurology, Cardiology, Haematology, Nephrology, Oncology, and Endocrinology. Families were invited to the Children’s Hospital of Lahore and provided with information related to genetic testing. After receiving genetic counseling and signing informed consent, families were invited for full anamnesis and clinical examination.

For the purpose of this research, all index cases and relatives tested during the period of 1 year (July 2018–July 2019) were included. Data from 1019 individuals, including 349 index cases and 670 relatives, were extracted from our database and individually curated.

### Ethics section

Written informed consents included: consent for genetic test related to the disease(s) of the patient, for secondary findings (unrelated to the main concern but clinically relevant, ACMG gene list^13^) and research findings (related to the main concern, but implicating genes not yet associated to human diseases). The consent form can be found as Supplementary information. Parents/guardians or adult patients signed the written consents. Written informed consent for publication of clinical data and images was obtained from the patients’ parents.

All analyses were performed in concordance to the provisions of the German Gene Diagnostic Act (Gendiagnostikgesetz). Ethical approval was granted by the Institutional Review Board (IRB)—Ethical committee from the Children’s hospital and the Institute of Child Health (Lahore), to cover the research aspects of the project.

### Genetic testing strategy

Blood samples were collected from the index and available relatives (dried blood spots on filter cards—CentoCard®), and sent to CENTOGENE laboratories (Rostock, Germany). Massive parallel sequencing was the most used method with ES being the most selected test, applied as first-line testing. In cases with clear phenotypes, the genetic testing strategy was based on clinical suspicion. Therefore, a relevant panel of genes was indicated if a specific disorder was suspected. Chromosomal microarray analysis (CMA) was mainly indicated in patients with multiple malformations. In cases with no genetic diagnosis, GS was performed as a reflex test. Additional tests, such as biochemical testing, MLPA, qPCR, and Sanger sequencing were performed to confirm initial sequencing findings where necessary. A summary of the genetic testing strategy is depicted in Supplementary Fig. 2.

Genetic testing was also offered to other close relatives, such as similarly affected siblings, or as part of predictive testing. DNA was extracted from dried blood spots on CentoCards® using standard, spin column-based methods.

### ES and GS

ES was performed as previously described^22^. In short, the Nextera Rapid Capture Exome Kit (Illumina, San Diego, CA) or the SureSelect Human All Exon kit (Agilent, Santa Clara, CA) were used for enrichment, and a HiSeq4000 (Illumina) instrument for the actual sequencing with the average coverage targeted to at least 100×. An in-house bioinformatics pipeline, including read alignment to GRCh37/hg19 genome assembly, variant calling, annotation and comprehensive variant filtering is applied. Our ES bioinformatics pipeline is based on the 1000 Genomes Project (1000G) data analysis—data pipeline and GATK best practice recommendations and is composed from widely used open source software projects. First, raw-sequencing reads are converted to standard fastq format using Illumina bcl2fastq software. Then short-reads are aligned to the GRCh37 (hg19) build of the human reference genome using bwa software with the mem algorithm. The alignments are converted to binary bam file format, sorted on the fly and de-duplicated without intermediate input–output operations to temporary files to achieve maximal performance. Afterward variant calling is performed on the secondary alignment files using three different variant callers (GATK HaplotypeCaller^26^, FreeBayes and SAMtools^27^). For GS, genomic DNA was fragmented by sonication, and Illumina adapters were ligated to generated fragments for subsequent sequencing on the HiSeqX platform (Illumina) to yield an average coverage depth of at least 30×. Raw sequence data analysis, including base calling, de-multiplexing, alignment to the hg19 human reference genome (GRCh37), and variant calling, was performed using the HiSeq Analysis Software v2.0 pipelines (Illumina, Inc., San Diego, CA). The short-reads were aligned to the GRCh37 (hg19) build of the human reference genome using Isaac aligner algorithm^28^. Variant calling was performed on the alignment files for SNVs and insertion–deletions (indels) using Starling Small Variant Caller^28^. Canvas^29^ and Manta^30^ were used to detect structural variants and CNVs. Variants were annotated using SnpEff^31^ and in-house bioinformatics tools^22^.

### Variant evaluation and classification

After variant annotations, filtering and prioritization were performed with an in-house developed tool. The system allows importing of the annotated variants and customized filtering taking into account variant and phenotype-related parameters (frequencies, zygosity, type of variant, HPO terms, mode of inheritance, among others). Trained scientists and human geneticists evaluated the clinical and genetic data. Relevant variants were considered based on compatibility with the suspected phenotype and disease mechanism. All provided clinical data, family history, consanguinity, disease onset/course, available test results, and clinical suspicions were considered. The clinical information was ‘translated’ into HPO terms, registered in our database, and applied for each analysis during variant filtration and prioritization since we previously detected relationship between the number of HPO terms and diagnostic results^22^. For the selected variants, mode of inheritance of the gene (OMIM®) and all relevant variant information were considered (zygosity, type of the variant, frequency in public databases – gnomAD, ExAc, and disease centered databases—HGMD^32^, CentoMD®^33^). For variants previously detected at CENTOGENE, clinical status and clinical information of the carrier individuals were evaluated as well, which aided in variant selection and final classification. Variant nomenclature followed standard recommendations^34,35^.

Selected candidate variants were classified according to published ACMG guidelines as pathogenic (P), likely pathogenic (LP), and variant of unknown significance (VUS)^36,37^. Likely benign and benign variants were excluded from reporting.

Interpretation of the findings was done in the clinical context, thus reports were issued as: (a) confirmed diagnosis, for P/LP variant(s) explaining the phenotype(s), (b) potential, for variants formally classified as VUS but with high evidence and compatible phenotype, and (c) unclear, for VUS compatible with the clinical phenotype.

### Other methods

Sanger sequencing, MLPA, qPCR, or CMA were performed depending on clinical suspicion or to confirm other tests results. For example, if a large heterozygous deletion was suspected based on ES data, a confirmation by an orthogonal method (e.g. qPCR) was performed. Forward and reverse primers were used for Sanger sequencing, on a 3730xl sequencer (Thermo Fisher Scientific, Waltham, MA). Genome-wide copy number variation + SNP analysis was performed using CytoScan® 750K Array and CytoScan® HD Array according to the manufacturer’s protocols (Thermo Fisher Scientific Inc.). Results were analyzed with the Chromosome Analysis Suite software (ChAS, Affymetrix, Inc., Santa Clara, CA). Analysis thresholds were as set at follows according to our validation to exclude false positive calls: heterozygous deletions with a minimum of 25 markers and/or a size >50 kb; homozygous deletions with at least five aberrant markers and a size >1 kb; duplications >200 kb; regions with absence of heterozygosity >3 Mb. MLPA® analyses were performed with commercially available kits according to the manufacturer’s instructions (MRC-Holland, Amsterdam, The Netherlands). MLPA reactions were run on an ABI 3730xl/3130xl DNA Analyzers (Applied Biosystems). To confirm CNV when no commercially available MLPA kit was available, we performed quantitative PCR assays (qPCR). When possible, in-house designs targeting 2–3 exons within the copy number variant and 1–2 additional fragments outside the alteration were used. Products were run on a LightCycler 480 II (Roche). Segregation of the variant(s) was evaluated in available family members.

The activities of the following enzymes were determined by fluorimetry in dried blood spots: alpha mannosidase, beta-hexosaminidase subunit A, total hexosaminidases, alpha-N-acetylgalactosaminidase, beta-glucocerebrosidase. The activities of the following enzymes were determined by liquid chromatography coupled with mass spectrometry in dried blood spots: acid sphingomyelinase, beta-galactosidase, N-acetylgalactosamine 6-sulfatase. The following biomarkers were quantified in dried blood spots using mass spectrometry: Glucosylsphingosine (Lyso-Gb1), Lyso-SM-509, Lyso-SM-465. All tests were clinically validated according to ISO 15189 guidelines.

### Clinical utility

Referring clinicians were retrospectively requested to fill a standard form to report on changes regarding clinical management after a genetic diagnosis was made based on: (i) new medical/surgical treatment implemented, (ii) changes in clinical management such as referral to other relevant medical specialties, changes in life style, avoidance of decompensating agents or special surveillance initiated, and (iii) prenatal diagnosis or predictive testing made possible.

### Reporting summary

Further information on research design is available in the Nature Research Reporting Summary linked to this article.

## Supplementary information

Supplementary material

Reporting Summary

Supplementary data 1

## Data Availability

The dataset generated and/or analyzed during the current study are available from the corresponding author on reasonable request. All pathogenic and likely pathogenic variants reported are submitted to ClinVar repository and accession numbers are provided in Supplementary Data 1.
