# Supplementary material for: Genomic testing in 1019 individuals from 349 Pakistani families results in high diagnostic yield and clinical utility
Source: NPJ Genom Med. 2020 Oct 5;5:44. doi: 10.1038/s41525-020-00150-z (PMC7536406; doi:10.1038/s41525-020-00150-z)
Supplement: Supplementary file 1 — Supplementary material [file 41525_2020_150_MOESM1_ESM.pdf]

Supplementary information

Genomic testing in more than 1,019 individuals from 349 Pakistani families results in high diagnostic yield and clinical utility

Huma Cheema[1*], Aida M. Bertoli-Avella[2*], Volha Skrahina[2], Muhammad Nadeem Anjum[1], Nadia Salman[1], Anjum Saeed[1], Christian Beetz[2], Jordi Perez-Lopez[2], Maria Eugenia Rocha[2], Salem Alawbathani, MD[2], Catarina Pereira, Msc[2], Marina Hovakimyan[2], Irene Patric[2], Omid Paknia[2] , Najim Ameziane[2], Claudia Cozma[2], Peter Bauer[2], Arndt Rolfs[2,3]

[1] Pediatric Department of Gastroenterology. Children's Hospital of Lahore Hospital, Lahore, Pakistan

[2] CENTOGENE AG, Rostock, Germany

[3] University of Rostock, Rostock, Germany

*Both authors contributed equally

Figure 1: Most frequently reported patient phenotypes based on HPOs corresponding to (A) metabolic/digestive system and (B) neurological phenotypes

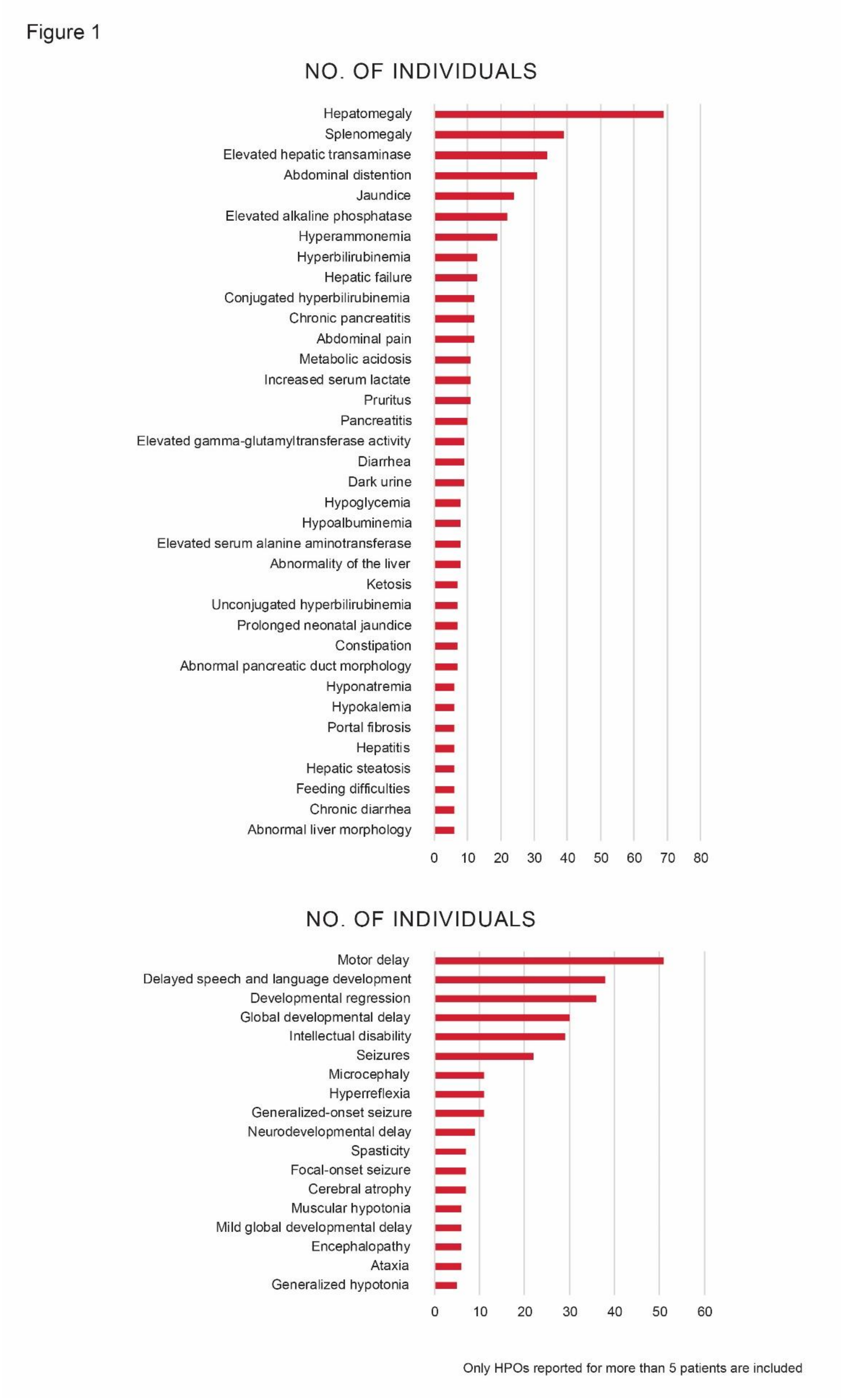

Figure 2. Summary of genetic testing performed related to 349 index cases. NGS panels were indicated if there was a strong clinical suspicion (e.g. lysosomal disease panel). Other testing included targeted gene analysis or CMA (1 case). NGS Exome sequencing was the most ordered test. Biochemical testing was done complementary to NGS findings.

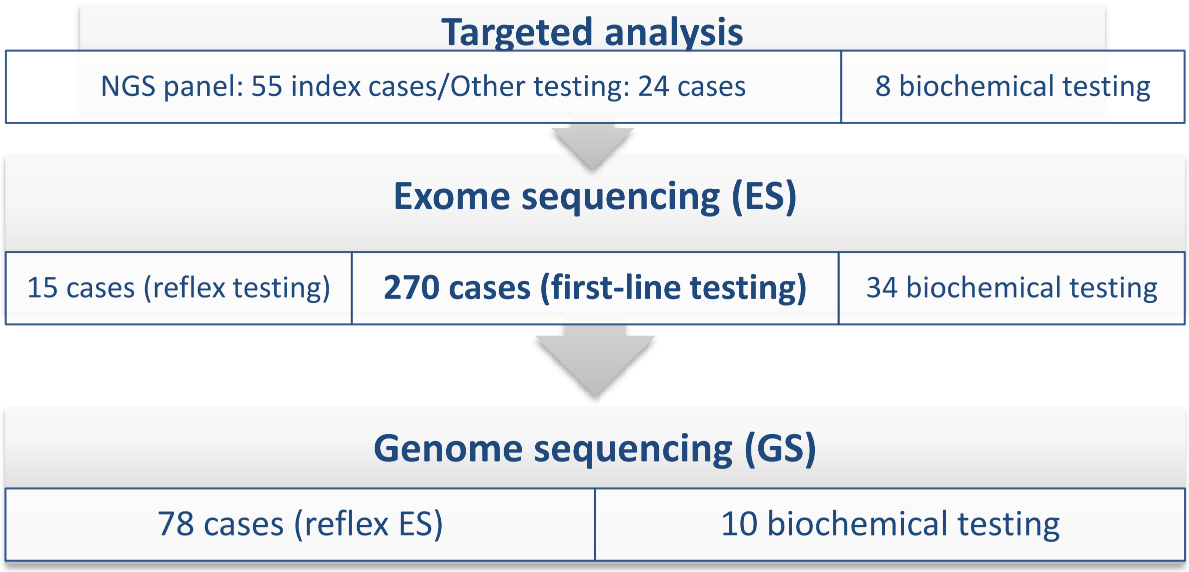

Table 1. Unique pathogenic and likely pathogenic variants (n=179) including SNV and CNV reported in this cohort. Note: in 15 families with deceased children, genetic testing was performed on the parents, heterozygous variants (for AR disorders) were detected with phenotype overlap with detected gene-related disease.

Gene	Ref.Seq. and nt change	Protein change	Zygocity	Disease (OMIM)	MOI
ABCB11	NM_003742.2:c.1156G>T	p.(Gly386*)	Het	Familial intrahepatic cholestasis type 2	AR
ABCB11	NM_003742.2:c.3382C>G	p.(Arg1128Gly)	Hom	Familial intrahepatic cholestasis type 2	AR
ABCB11	NM_003742.2:c.3691C>T	p.(Arg1231Trp)	Hom	Familial intrahepatic cholestasis type 2	AR
ABCB11	NC_000002.11(NM_003742.2):c.908+1G>C	Splicing	Hom	Familial intrahepatic cholestasis type 2	AR
ABCB11	NC_000002.11(NM_003742.2):c.2178+2T>C	Splicing	Hom	Familial intrahepatic cholestasis type 2	AR
ABCB4	NM_018849.2:c.1783C>T	p.(Arg595*)	Hom	Familial intrahepatic cholestasis type 3	AR
ABCB4	NM_018849.2:c.874A>T	p.(Lys292*)	Hom	Familial intrahepatic cholestasis type 3	AR
ABCC8	NM_001351295.1:c.526dup	p.(Val176Glyfs*96)	Hom	Familial hyperinsulinemic hypoglycemia type 1	AR
ABCC9	NM_005691.2:c.3190G>C	p.(Ala1064Pro)	Het	Cantu syndrome	AD
ABHD5	NM_016006.5:c.730dup	p.(Thr244Asnfs*10)	Hom	Chanarin-Dorfman syndrome	AR
ADA2	chr22:17687819-17690721del (exons 2-3 deletion)		Hom	Polyarteritis nodosa	AR
AGL	NM_000028.2:c.567dup	p.(Arg190*)	Hom	Glycogen storage disease type IIIA/IIIB	AR
AGL	NM_000028.2:c.100C>T	p.(Arg34*)	Hom	Glycogen storage disease type IIIA/IIIB	AR
AGL	NM_000028.2:c.1078C>T	p.(His360Tyr)	Hom	Glycogen storage disease type IIIA/IIIB	AR
AGL	NM_000028.2:c.1497_1500dup	p.(Asp501Argfs*15)	Hom	Glycogen storage disease type IIIA/IIIB	AR
AGL	NM_000028.2:c.753_756del	p.(Asp251Glufs*23)	Hom	Glycogen storage disease type IIIA/IIIB	AR
AGL	NM_000642.2:c.2399dup	p.(Asp801Argfs*7)	Hom	Glycogen storage disease type IIIA/IIIB	AR
AGXT	NM_000030.2:c.302T>C	p.(Leu101Pro)	Hom	Type I primary hyperoxaluria	AR
ALPL	NM_000478.5:c.571G>A	p.(Glu191Lys)	Hom	Odontohypophosphatasia	AR
ALS2	NM_020919.3:c.4753_4754dup	p.(Ser1585Argfs*34)	Hom	Juvenile amyotrophic lateral sclerosis type 2	AR
ANK1	NM_001142446.1:c.1816del	p.(Leu606Cysfs*64)	Het	Spherocytosis type 1	AD
ASL	NM_000048.3:c.545G>A	p.(Arg182Gln)	Het	Argininosuccinic aciduria	AR
ASPA	NM_000049.2:c.162C>A	p.(Asn54Lys)	Hom	Canavan disease	AR
ASPA	NM_000049.2:c.692A>G	p.(Tyr231Cys)	Hom	Canavan disease	AR
ATM	NM_000051.3:c.3503dup	p.(Cys1168Trpfs*1 1)	Hom	Ataxia-telangiectasia	AR
ATM	NM_000051.3:c.3680_3683del	p.(Thr1227Asnfs*1 2)	Hom	Ataxia-telangiectasia	AR
ATP6V0A 4	NM_020632.2:c.2257C>T	p.(Gln753*)	Hom	Distal renal tubular acidosis	AR
ATP6V0A 4	chr7:138400344-138400688del (exon 20 deletion)		Hom	Distal renal tubular acidosis	AR
ATP7B	NM_000053.2:c.3182G>A	p.(Gly1061Glu)	Hom	Wilson disease	AR
ATP7B	NM_000053.2:c.3305T>C	p.(Ile1102Thr)	Hom	Wilson disease	AR

ATP7B	NM_000053.3:c.3301G>A	p.(Gly1101Arg)	Hom	Wilson disease	AR
ATP7B	NM_000053.3:c.856C>T	p.(Gln286*)	Hom	Wilson disease	AR
ATP8B1	NM_005603.4:c.1804C>T	p.(Arg602*)	Hom	Familial intrahepatic cholestasis type 1	AR
ATP8B1	NM_005603.4:c.589_592delinsCTCCA	p.(Gly197Leufs*10)	Hom	Familial intrahepatic cholestasis type 1	AR
BMPR1A	NM_004329.2:c.44_47del	p.(Leu15Serfs*20)	Het	Juvenile polyposis syndrome	AD
BRCA1	NM_007300.3:c.1921dup	p.(Ile641Asnfs*2)	Het	Familial breast-ovarian cancer type 1	AD
BTD	NM_001281723.2:c.104_110delinsTCC	p.(Cys35Phefs*36)	Hom	Biotinidase deficiency	AR
BTD	NM_001281723.2:c.1336G>C	p.(Asp446His)	Het	Biotinidase deficiency	AR
BTD	NM_001281723.2:c.1514_1518del	p.(Gly505Aspfs*6)	Hom	Biotinidase deficiency	AR
BTK	NM_001287344.1:c.402T>A	p.(Tyr134*)	Hem	Agammaglobulinemia, X-linked type 1	XL
BTK	NM_001287344.1:c.865C>T	p.(Arg289*)	Hem	Agammaglobulinemia, X-linked type 1	XL
C1R	NM_001354346.1:c.1469_1470dup	p.(Trp491Profs*55)	Het	Ehlers-Danlos syndrome periodontal type 1	AD
CA5A	NM_001739.1:c.580C>T	p.(Gln194*)	Hom	Carbonic anhydrase VA deficiency	AR
CBS	NM_000071.2:c.434C>T	p.(Pro145Leu)	Hom	Homocystinuria	AR
CD36	chr7:80279064-80356762del (exon 4 to 15 deletion)		Hom	Platelet glycoprotein IV deficiency	AR
CDC42	NM_001039802.1:c.191A>G	p.(Tyr64Cys)	Het	Takenouchi-Kosaki syndrome	AD
CFTR	NM_000492.3:c.1521_1523del	p.(Phe508del)	Hom	Cystic fibrosis	AR
CFTR	NM_000492.3:c.1705T>G	p.(Tyr569Asp)	Het	Cystic fibrosis	AR
CFTR	NM_000492.3:c.2758G>A	p.(Val920Met)	Het	Cystic fibrosis	AR
CFTR	NM_000492.3:c.3209G>A	p.(Arg1070Gln)	Hom	Cystic fibrosis	AR
CHRNE	NM_000080.3:c.1367_1369del	p.(Asn456del)	Hom	Congenital myasthenic syndrome type 4B, fast-channel	AR
CLN6	NM_017882.2:c.316dup	p.(Arg106Profs*26)	Hom	Neuronal ceroid lipofuscinosis type 6	AR
CNGA3	NM_001298.2:c.1306C>T	p.(Arg436Trp)	Hom	Achromatopsia type 2	AR
COL17A1	NM_000494.3:c.3539dup	p.(Gly1181Argfs*61)	Hom	Junctional epidermolysis bullosa	AR
COL4A4	NM_000092.4:c.3933C>G	p.(Tyr1311*)	Hom	Alport syndrome type 2	AR
CTNS	NM_001031681.2:c.771_793del	p.(Gly258Serfs*30)	Hom	Cystinosis	AR
CYP21A2	NM_000500.5:c.955C>T	p.(Gln319*)	Hom	Congenital adrenal hyperplasia, due to 21-hydroxylase deficiency	AR
CYP27A1	NC_000002.11(NM_000784.3):c.1184+1G>A	Splicing	Hom	Cerebrotendinous xanthomatosis	AR
DBT	NM_001918.2:c.939G>C	p.(Lys313Asn)	Hom	Maple syrup urine disease type II	AR
DCDC2	chr6:24205090-24205614del (exon 9 deletion)		Hom	Neonatal sclerosing cholangitis	AR
DDC	NM_000790.3:c.1040G>A	p.(Arg347Gln)	Het	Aromatic L-amino acid decarboxylase deficiency	AR
DLD	NM_000108.3:c.685G>T	p.(Gly229Cys)	Hom	Dihydrolipoamide dehydrogenase deficiency	AR
DMD	NM_004006.2:c.1087C>T	p.(Gln363*)	Hem	Duchenne muscular dystrophy	XL
DPYD	NM_000110.3:c.1475C>T	p.(Ser492Leu)	Hom	Dihydropyrimidine dehydrogenase deficiency	AR

DSG2	NC_000018.9(NM_001943.4):c.45+1G>C	Splicing	Het	Arrhythmogenic right ventricular dysplasia type 10	AD
EPCAM	NM_002354.2:c.13C>T	p.(Gln5*)	Hom	Diarrhea with congenital tufting enteropathy type 5	AR
EPCAM	NM_002354.2:c.439G>T	p.(Glu147*)	Hom	Diarrhea with congenital tufting enteropathy type 5	AR
ETFDH	NM_004453.2:c.1448C>T	p.(Pro483Leu)	Het	Acyl-CoA dehydrogenase deficiency, glutaric acidemia type II	AR
ETHE1	NM_014297.3:c.487C>T	p.(Arg163Trp)	Hom	Ethylmalonic encephalopathy	AR
FA2H	NM_024306.4:c.806G>A	p.(Arg269His)	Hom	Spastic paraplegia type 35	AR
FAH	NC_000015.9(NM_000137.2):c.1062+5G>A	Splicing	Hom	Tyrosinemia type I	AR
FAH	NM_000137.2:c.192G>T	p.(Gln64His)	Hom	Tyrosinemia type I	AR
FAH	NM_000137.2:c.974C>T	p.(Thr325Met)	Hom	Tyrosinemia type I	AR
FBN1	NM_000138.4:c.5636G>A	p.(Gly1879Asp)	Het	Marfan syndrome	AD
FBP1	NM_000507.3:c.778G>A	p.(Gly260Arg)	Hom	Fructose-1,6-bisphosphatase deficiency	AR
FBP1	NM_000507.3:c.841G>A	p.(Glu281Lys)	Hom	Fructose-1,6-bisphosphatase deficiency	AR
FBP1	NM_001127628.1:c.472C>T	p.(Arg158Trp)	Hom	Fructose-1,6-bisphosphatase deficiency	AR
FUCA1	NM_000147.4:c.691G>A	p.(Gly231Arg)	Hom	Fucosidosis	AR
G6PC	chr17:41062931-41063443del (exon 5 deletion)		Hom	Glycogen storage disease type 1A	AR
G6PC	NM_000151.3:c.61del	p.(Val21*)	Hom	Glycogen storage disease type 1A	AR
G6PD	NM_000402.3:c.653C>T	p.(Ser218Phe)	Hem	G6PD deficient hemolytic anemia	XL
G6PD	NM_000402.3:c.961G>A	p.(Val321Met)	Hem	G6PD deficient hemolytic anemia	XL
GALNS	**NM_001323544.1:c.470C>T**	p.(Pro157Leu)	Hom	Mucopolysaccharidosis type IVA	AR
GALNS	**NM_001323544.1:c.516C>G**	p.(His172Gln)	Hom	Mucopolysaccharidosis type IVA	AR
GALT	NM_000155.2:c.563A>G	p.(Gln188Arg)	Hom	Galactosemia	AR
GALT	NC_000009.11(NM_000155.3):c.377+1G>T	Splicing	Hom	Galactosemia	AR
GBA	**NM_000157.3:c.1448T>C**	p.(Leu483Pro)	Hom	Gaucher disease	AR
GLB1	**NM_001317040.1:c.1025_1026delAT**	p.(Tyr342fs)	Hom	GM1-gangliosidosis	AR
GLB1	**NM_001317040.1:c.1399C>T**	p.(Arg467Trp)	Hom	GM1-gangliosidosis	AR
GLB1	**NM_001317040.1:c.1398C>G**	p.(Tyr466*)	Hom	GM1-gangliosidosis	AR
GNB5	NM_016194.3:c.1032C>A	p.(Tyr344*)	Hom	Intellectual developmental disorder with cardiac arrhythmia	AR
GNPTAB	NM_024312.4:c.3503_3504del	p.(Leu1168Glnfs*5)	Hom	Mucolipidosis type II	AR
HBB	NM_000518.4:c.27dup	p.(Ser10Valfs*14)	Het	Beta-thalassemia	AD
HBB	NM_000518.4:c.51del	p.(Lys18Argfs*2)	Het	Beta-thalassemia	AD
HEXA	**NM_000520.4:c.1274_1277dup**	p.(Tyr427Ilefs*5)	Hom	Tay-Sachs disease/GM2-gangliosidosis	AR
HEXA	**NM_000520.5:c.902T>G**	p.(Met301Arg)	Hom	Tay-Sachs disease/GM2-gangliosidosis	AR
HEXA	**NM_000520.5:c.109T>A**	p.(Tyr37Asn)	Hom	Tay-Sachs disease/GM2-gangliosidosis	AR
HEXB	**NM_000521.3:c.850C>T**	p.(Arg284*)	Hom	Sandhoff disease	AR
HEXB	**NM_000521.3:c.1597C>T**	p.(Arg533Cys)	Hom	Sandhoff disease	AR

IDUA	chr4:996510-998191del (exons 10-14 deletion)		Hom	Mucopolysaccharidosis type I	AR
IGHM	ENST00000390559.2:c.750dup	p.(Thr251Hisfs*3)	Hom	Agammaglobulinemia type 1	AR
IL21R	NC_000016.9(NM_181079.4):c.418+1G>C	Splicing	Hom	Immunodeficiency type 56	AR
ITPA	NM_033453.3:c.136_138delinsTAA	p.(Gln46*)	Hom	Early infantile epileptic encephalopathy type 35	AR
IVD	NM_002225.3:c.899C>T	p.(Ala300Val)	Hom	Isovaleric acidemia	AR
L2HGDH	chr14:50731869-50732220del (exon 9 deletion)		Hom	L-2-hydroxyglutaric aciduria	AR
LPL	NC_000008.10(NM_000237.2):c.1139+1G>A	Splicing	CH	Lipoprotein lipase deficiency	AR
LPL	NM_000237.2:c.784C>T	p.(Gln262*)	CH	Lipoprotein lipase deficiency	AR
LPL	NM_000237.2:c.987C>A	p.(Tyr329*)	Hom	Lipoprotein lipase deficiency	AR
LPL	NM_000237.2:c.1160_1161insT	p.(Lys387Asnfs*26)	Hom	Lipoprotein lipase deficiency	AR
LRBA	NC_000004.11(NM_006726.4):c.4159-1G>T	Splicing	Hom	Immunodeficiency type 8	AR
LRBA	NM_006726.4:c.4333C>T	p.(Arg1445*)	Hom	Immunodeficiency type 8	AR
MAN2B1	**NC_000019.9(NM_000528.3):c.1644+4A>G**	Splicing	Hom	Alpha-Mannosidosis	AR
MEFV	NM_000243.2:c.688G>A	p.(Glu230Lys)	Het	Familial Mediterranean fever	AD
MLH1	NM_000249.2:c.1528_1532delinsACTAGTTTG	p.(Gln510Thrfs*6)	Het	Hereditary nonpolyposis colorectal cancer type 2	AD
MMAA	NM_172250.2:c.72C>A	p.(Tyr24*)	Hom	Methylmalonic aciduria of the cblA complementation type	AR
MMACHC	NM_015506.2:c.394C>T	p.(Arg132*)	Hom	cblC type of combined methylmalonic aciduria and homocystinuria	AR
MPV17	NM_002437.4:c.293C>T	p.(Pro98Leu)	Hom	Mitochondrial DNA depletion syndrome type 6	AR
NAGLU	**NM_000263.3:c.1336G>A**	p.(Glu446Lys)	Hom	Mucopolysaccharidosistype IIIB	AR
NAGLU	**NM_000263.3:c.291T>G**	p.(Cys97Trp)	Hom	Mucopolysaccharidosistype IIIB	AR
NAGLU	**NM_000263.3:c.701G>C**	p.(Arg234Pro)	Hom	Mucopolysaccharidosistype IIIB	AR
NALCN	NM_001350748.1:c.883del	p.(Arg295Valfs*19)	Het	Congenital contractures of the limbs and face, hypotonia and developmental delay	AD
NDUFV1	NM_007103.3:c.1268C>T	p.(Thr423Met)	Hom	Mitochondrial complex I deficiency nuclear type 4	AR
NPC1	**NM_000271.4:c.1097C>G**	p.(Ser366*)	Hom	Niemann-Pick disease type C1	AR
NPC1	**NM_000271.4:c.2608T>A**	p.(Ser870Thr)	Hom	Niemann-Pick disease type C1	AR
NPC1	**NM_000271.4:c.2978dup**	p.(Asp994Argfs*13)	Hom	Niemann-Pick disease type C1	AR
NPC1	**NM_000271.4:c.3503G>A**	p.(Cys1168Tyr)	Hom	Niemann-Pick disease type C1	AR
NPC1	**NM_000271.4:c.3020C>T**	p.(Pro1007Leu)	Hom	Niemann-Pick disease type C1	AR
NPHP1	chr2:110827496-110962791del (whole gene deletion)		Hom	Joubert syndrome type 4	AR
OXCT1	NC_000005.9(NM_000436.3):c.1339-2A>G	Splicing	Hom	Succinyl-CoA:3-oxoacid-CoA transferase deficiency	AR
PANK2	chr20:3844158-3898983del (exons 1-4 deletion)		Hom	Neurodegeneration with brain iron accumulation type 1	AR

PEX1	NC_000007.13(NM_000466.2):c.2926+2T>C	Splicing	Het	Peroxisome biogenesis disorder type 1A	AR
PEX1	NM_000466.2:c.2T>C	p:?	Hom	Peroxisome biogenesis disorder type 1A	AR
PHKG2	NM_000294.2:c.553C>T	p.(Arg185*)	Hom	Glycogen storage disease type IXc	AR
PHKG2	NM_000294.2:c.454C>T	p.(Arg152*)	Hom	Glycogen storage disease type IXc	AR
PLA2G6	NC_000022.10(NM_003560.2):c.1742+2T>G	Splicing	Hom	Neurodegeneration with brain iron accumulation 2B	AR
PRF1	NM_001083116.1:c.921del	p.(His308Thrfs*22)	Het	Familial hemophagocytic lymphohistiocytosis type 2	AR
PRG4	NM_005807.4:c.3462_3465del	p.(Thr1155Leufs*7)	Hom	Camptodactyly-arthropathy-coxa vara-pericarditis syndrome	AR
PRSS1	chr7:141443350-142460881dup (gene duplication)		Het	Hereditary pancreatitis	AD
PRSS1	NM_002769.4:c.365G>A	p.(Arg122His)	Het	Hereditary pancreatitis	AD
PRSS1	chr7:142456977-142460891dup (exons 1-5 duplication)		Het	Hereditary pancreatitis	AD
PTPN11	NM_001330437.1:c.205G>A	p.(Glu69Lys)	Het	Noonan syndrome type 1	AD
RELB	NM_006509.3:c.919C>T	p.(Arg307*)	Hom	Immunodeficiency type 53	AR
SCN5A	NM_001099404.1:c.6046G>A	p.(Val2016Met)	Het	Brugada syndrome type 1 / Long QT syndrome type 3	AD
SI	NM_001041.3:c.2401G>T	p.(Glu801*)	Hom	Congenital sucrose-isomaltase deficiency	AR
SLC19A3	NM_025243.3:c.482_483del	p.(Leu161Cysfs*63)	Hom	Thiamine metabolism dysfunction syndrome type 2	AR
SLC22A5	NM_001308122.1:c.1481C>T	p.(Ser494Phe)	Hom	Primary carnitine deficiency	AR
SLC25A15	NM_014252.3:c.564C>G	p.(Phe188Leu)	Hom	Hyperornithinemia-hyperammonemia-homocitrullinuria syndrome	AR
SLC25A15	NM_014252.3:c.44C>T	p.(Ala15Val)	Hom	Hyperornithinemia-hyperammonemia-homocitrullinuria syndrome	AR
SLC34A3	NC_000009.11(NM_001177316.1):c.448+1G>A	Splicing	Hom	Hypophosphatemic rickets with hypercalciuria	AR
SLC37A4	NM_001164278.1:c.92_94del	p.(Phe31del)	Hom	Glycogen storage disease type 1B	AR
SLC37A4	NM_001164278.1:c.169_175del	p.(Ser57Leufs*16)	Hom	Glycogen storage disease type 1B	AR
SLC37A4	NM_001164278.1:c.936dup	p.(Val313Serfs*13)	Hom	Glycogen storage disease type 1B	AR
SLC5A1	NM_000343.3:c.187C>T	p.(Arg63*)	Hom	Glucose/galactose malabsorption	AR
SMN1	chr5:70241893-70247818del (exon 7-8 deletion)		Hom	Spinal muscular atrophy type 1	AR
SMN1	chr5: 70241893-70247818del (exon 7-8 deletion)		Het	Spinal muscular atrophy type 1	AR
SMPD1	**NM_000543.4:c.1624C>T**	p.(Arg542*)	Het	Niemann-Pick disease type A/B	AR
SMPD1	**NM_000543.4:c.1382_1383del**	p.(His461Argfs*3)	Hom	Niemann-Pick disease type A/B	AR
SMPD1	**NM_000543.4:c.1493G>A**	p.(Arg498His)	Hom	Niemann-Pick disease type A/B	AR
SMPD1	**NM_000543.4:c.314T>C**	p.(Leu105Pro)	Hom	Niemann-Pick disease type A/B	AR
SMPD1	**NM_000543.4:c.748A>C**	p.(Ser250Arg)	Hom	Niemann-Pick disease type A/B	AR
SOD1	NM_000454.4:c.377A>C	p.(Asp126Ala)	Hom	Amyotrophic lateral sclerosis type 1	AR

STXBP2	NC_000019.9(NM_001272034.1):c.1280-1G>C	Splicing	Hom	Familial hemophagocytic lymphohistiocytosis type 5	AR
TANGO2	NM_001322141.1:c.541C>T	p.(Arg181*)	Hom	Metabolic crises with rhabdomyolysis, cardiac arrhythmias, and neurodegeneration	AR
TJP2	NC_000009.11(NM_001170416.1):c.332+1G>A	Splicing	Hom	Progressive familial intrahepatic cholestasis type 4	AR
TJP2	NM_001170416.1:c.2420del	p.(Leu807fs)	Hom	Progressive familial intrahepatic cholestasis type 4	AR
TMPRSS15	NM_002772.2:c.2294del	p.(Leu765fs)	Hom	Enterokinase deficiency	AR
TMPRSS15	NM_002772.2:c.2325del	p.Lys775fs	Hom	Enterokinase deficiency	AR
TNNT2	NM_001276345.1:c.547C>T	p.(Arg183Trp)	Het	Dilated cardiomyopathy type 1D/ Left ventricular noncompaction type 6	AD
TRMU	NM_018006.4:c.835G>A	p.(Val279Met)	Hom	Transient infantile liver failure	AR
*UFM1***	NC_000013.10(NM_016617.2):c.-155_-153del*	Splicing	Hom	Hypomyelinating leukodystrophytype type 14	AR
UGT1A1	NM_000463.2:c.1021C>T	p.(Arg341*)	Hom	Crigler-Najjar syndrome type 1	AR
UGT1A1	NM_000463.2:c.1091C>T	p.(Pro364Leu)	Het	Crigler-Najjar syndrome type 1	AR
VWF	NM_000552.3: c.3931C>T	p.(Gln1311*)	Hom (gene conversion)	von Willebrand disease	AR
WDR62	NM_001083961.1:c.1531G>A	p.(Asp511Asn)	Hom	Primary microcephaly type 2	AR
XIAP	chrX:123019502-123022578del (exons 2-3 deletion)		Hem	Lymphoproliferative syndrome type 2	XL
ZBTB24	NM_014797.2:c.1369C>T	p.(Arg457*)	Hom	Immunodeficiency-centromeric instability-facial anomalies syndrome type 2	AR
Chr. 11	chr11:104288964-134937416dup		Het	Distal trisomy 11q	n/a
Chr. 22	chr22:21514655-22986816del		Het	Distal chromosome 22q11.2 deletion syndrome	n/a
Chr. X	chrX:168551-155233098del		Het	Turner syndrome	n/a

*Variant is published as as NM_001286704.1:c.-273_-271del. Variants **in bold** are presented also in table 2 (with confirmatory biochemical testing).

Hom: homozygous, Het: heterozygous, Hem; Hemizygous, CH: compound heterozygous, Ref. Seq: reference sequence, nt: nucleotide, MOI: Mode of inheritance, AR: autosomal recessive, AD: autosomal dominant, XL: X-linked. For diseases with both AD and AR MOI, the MOI corresponding to the patient has been annotated.

Table 2. Diseases diagnosed in this cohort and clinical impact in these patients.

Disease	Mode of inheritance	OMIM/ORPHA number	New therapy implemented	Referral to other specialties
Achromatopsia type 2	AR	216900		
Acyl-CoA dehydrogenase deficiency, glutaric acidemia type II	AR	231680	Yes	
Agammaglobulinemia type 1	AR	601495	Yes	
Agammaglobulinemia, X-linked type 1	XL	300755	Yes	
Alpha-Mannosidosis	AR	248500		Yes
Alport syndrome type 2	AR	203780	Yes	Yes
Amyotrophic lateral sclerosis type 1	AR	105400	Yes	Yes
Argininosuccinic aciduria	AR	207900	Yes	
Aromatic L-amino acid decarboxylase deficiency	AR	608643	Yes	
Arrhythmogenic right ventricular dysplasia type 10	AD	610193		
Ataxia-telangiectasia	AR	208900		
Beta-thalassemia	AD	613985		Yes
Biotinidase deficiency	AR	253260	Yes	
Brugada syndrome type 1 / Long QT syndrome type 3	AD	601144/603830	Yes	Yes
Camptodactyly-arthropathy-coxa vara-pericarditis syndrome	AR	208250		
Canavan disease	AR	271900		Yes
Cantu syndrome	AD	239850		
Carbonic anhydrase VA deficiency	AR	114761		
cblC type of combined methylmalonic aciduria and homocystinuria	AR	277400		
Cerebrotendinous xanthomatosis	AR	213700	Yes	
Chanarin-Dorfman syndrome	AR	275630		
Congenital adrenal hyperplasia, due to 21-hydroxylase deficiency	AR	201910	Yes	Yes
Congenital contractures of the limbs and face, hypotonia and developmental delay	AD	616266		
Congenital myasthenic syndrome type 4B, fast-channel	AR	616324		
Congenital sucrose-isomaltase deficiency	AR	222900	Yes	
Cystic fibrosis	AR	219700		Yes
Cystinosis	AR	219800	Yes	Yes
Diarrhea with congenital tufting enteropathy type 5	AR	613217	Yes	
Dihydrolipoamide dehydrogenase deficiency	AR	246900	Yes	
Dihydropyrimidine dehydrogenase deficiency	AR	274270	Yes	
Dilated cardiomyopathy type 1D/ Left ventricular noncompaction type 6	AD	601494/601494	Yes	Yes
Distal chromosome 22q11.2 deletion syndrome	n/a	611867		
Distal renal tubular acidosis	AR	602722	Yes	Yes
Distal trisomy 11q	n/a	ORPHA:96103		
Duchenne muscular dystrophy	XL	310200		Yes
Early infantile epileptic encephalopathy type 35	AR	616647		Yes
Ehlers-Danlos syndrome periodontal type 1	AD	130080		
Enterokinase deficiency	AR	226200	Yes	
Ethylmalonic encephalopathy	AR	602473		Yes
Familial breast-ovarian cancer type 1	AD	604370	Yes	Yes
Familial hemophagocytic lymphohistiocytosis type 2	AR	603553	Yes	
Familial hemophagocytic lymphohistiocytosis type 5	AR	613101	Yes	

Familial hyperinsulinemic hypoglycemia type 1	AR/AD	256450	Yes	
Familial intrahepatic cholestasis type 1	AR	211600	Yes	
Familial intrahepatic cholestasis type 2	AR	601847	Yes	
Familial intrahepatic cholestasis type 3	AR	602347	Yes	
Familial Mediterranean fever	AD/AR	249100	Yes	
Fructose-1,6-bisphosphatase deficiency	AR	229700	Yes	
Fucosidosis	AR	230000		
G6PD deficient hemolytic anemia	XL	300908	Yes	Yes
Galactosemia	AR	230400	Yes	
Gaucher disease	AR	230800, 23090, 231000	Yes	
Gilbert syndrome / Crigler-Najjar syndrome	AR	143500, 218800		Yes
Glucose/galactose malabsorption	AR	606824	Yes	
Glycogen storage disease type IXc	AR	613027	Yes	
Glycogen storage disease type 1A	AR	232200	Yes	
Glycogen storage disease type 1B	AR	232220	Yes	
Glycogen storage disease type IIIA/IIIB	AR	232400	Yes	
GM1-gangliosidosis	AR	230500		Yes
Hereditary nonpolyposis colorectal cancer type 2	AD	609310	Yes	
Hereditary pancreatitis	AD	167800		
Homocystinuria	AR	236200	Yes	
Hyperornithinemia-hyperammonemia-homocitrullinuria syndrome	AR	238970	Yes	
Hypomyelinating leukodystrophytype type 14	AR	617899		Yes
Hypophosphatemic rickets with hypercalciuria	AR	241530	Yes	
Immunodeficiency type 53	AR	617585	Yes	
Immunodeficiency type 56	AR	615207	Yes	Yes
Immunodeficiency type 8	AR	614700	Yes	
Immunodeficiency-centromeric instability-facial anomalies syndrome type 2	AR	614069	Yes	
Intellectual developmental disorder with cardiac arrhythmia	AR	617173		Yes
Isovaleric acidemia	AR	243500	Yes	
Joubert syndrome type 4	AR	609583		
Junctional epidermolysis bullosa	AR	226650		Yes
Juvenile polyposis syndrome	AD	174900	Yes	Yes
Juvenile amyotrophic lateral sclerosis type 2	AR	205100		Yes
L-2-hydroxyglutaric aciduria	AR	236792	Yes	
Lipoprotein lipase deficiency	AR	238600	Yes	
Long QT syndrome type 1	AD	192500	Yes	
Lymphoproliferative syndrome type 2	XL	300635	Yes	
Maple syrup urine disease type II	AR	248600	Yes	
Marfan syndrome	AD	154700		Yes
Metabolic crises with rhabdomyolysis, cardiac arrhythmias, and neurodegeneration	AR	616878	Yes	
Methylmalonic aciduria of the cblA complementation type	AR	251100	Yes	
Mitochondrial complex I deficiency nuclear type 4	AR	618225		
Mitochondrial DNA depletion syndrome type 6	AR	256810		
Mucolipidosis type II	AR	252500		
Mucopolysaccharidosis type I	AR	252800		
Mucopolysaccharidosis type IVA	AR	253000		Yes
Mucopolysaccharidosistype IIIB	AR	252920	Yes	Yes
Neonatal sclerosing cholangitis	AR	617394	Yes	
Neurodegeneration with brain iron accumulation type 1	AR	234200		Yes

Neurodegeneration with brain iron accumulation 2B	AR	610217		Yes
Neuronal ceroid lipofuscinosis type 6	AR	601780		Yes
Niemann-Pick disease type A/B	AR	257200/607616		Yes
Niemann-Pick disease type C1	AR	257220		
Noonan syndrome type 1	AD	163950		Yes
Odontohypophosphatasia	AD/AR	146300		
Peroxisome biogenesis disorder type 1A	AR	214100		Yes
Platelet glycoprotein IV deficiency	AR	608404		
Polyarteritis nodosa	AR	615688	Yes	Yes
Primary carnitine deficiency	AR	212140	Yes	
Primary microcephaly type 2	AR	604317		Yes
Progressive familial intrahepatic cholestasis type 4	AR	615878	Yes	
Sandhoff disease	AR	268800		Yes
Spastic paraplegia type 35	AR	612319		Yes
Spherocytosis type 1	AD/AR	182900	Yes	Yes
Spinal muscular atrophy types 1	AR	253300		Yes
Succinyl-CoA:3-oxoacid-CoA transferase deficiency	AR	245050	Yes	Yes
Takenouchi-Kosaki syndrome	AD	616737		
Tay-Sachs disease/GM2-gangliosidosis	AR	272800		Yes
Thiamine metabolism dysfunction syndrome type 2	AR	607483	Yes	Yes
Transient infantile liver failure	AR	613070	Yes	
Turner syndrome	n/a	ORPHA:881		Yes
Type I primary hyperoxaluria	AR	259900	Yes	
Tyrosinemia type I	AR	276700	Yes	
von Willebrand disease	AR	193400, 613554, 277480	Yes	Yes
Wilson disease	AR	277900	Yes	

AR: autosomal recessive, AD: autosomal dominant, XL: X-linked, n/a: not applicable

Table 3. Dual diagnoses: patients' clinical presentations and pathogenic/likely pathogenic variants identified.

Patient	Clinical presentation	Zyg	Ref. Seq: nt change	Protein change	Disease 1, MOI	Zyg .	Ref. Seq: nt change	Protein change	Disease 2, MOI
1307875	Chronic pancreatitis, pancreatic pseudocyst	Het	*SPINK1* NM_003122.3:c.101A>G	p.(Asn34Ser)	Chronic pancreatitis, AD/AR	Hom	*CBS* NM_000071.2:c.434C>T	p.(Pro145Leu)	Homocystinuria, AR
1333978	Neurodegenerative symptoms, dysarthria, seizures, ID	Hom	*HEXA* NM_001318825.1:c.109T>A	p.(Tyr37Asn)	Tay-Sachs disease, AR	Het	*C1R* NM_001354346.1: c.1469_1470dup	p.(Trp491Profs *55)	Ehlers-Danlos syndrome periodontal type 1, AD
1361835	Abdominal distention, acholic stools, aciduria, hepatomegaly, inability to walk, ketosis, lactic acidosis, methylmalonic acidemia, neurodevelopmental delay	Hom	NM_014297.3(*ETHE1*):c.487C>T	p.(Arg163Trp)	Ethylmalonic encephalopathy, AR	Hem	*G6PD* NM_000402.3:c.961G>A	p.(Val321Met)	G6PD deficiency, XL
1362079	Aggressive behavior, anemia, anisocytosis, beaking of vertebral bodies, behavioral abnormality, coarse facial features, diarrhea, dysostosis multiplex, failure to thrive, hepatomegaly, hyperactivity, hypercholesterolemia, hypertriglyceridemia, hypochromic microcytic anemia, ID, poikilocytosis, progressive neurologic deterioration, recurrent lower respiratory tract infections, splenomegaly	Hom	*ALPL* NM_000478.5:c.571G>A	p.(Glu191Lys)	Infantile hypophosphatasia, AR	Hom	*FUCA1* NM_000147.4:c.691G>A	p.(Gly231Arg)	Fucosidosis, AR
1390518	Abnormal glucose-6-phosphate dehydrogenase level, abnormal levels of alpha-fetoprotein, elevated alkaline phosphatase, elevated hepatic transaminase, hepatomegaly, hyperbilirubinemia, hypercholesterolemia, hypoalbuminemia, jaundice, neonatal onset, osteopenia, pruritus, splenomegaly, unconjugated hyperbilirubinemia	Hom	*ABCB4* NM_018849.2:c.874A>T	p.(Lys292*)	Familial intrahepatic cholestasis type 3, AR	Hem	*G6PD* NM_000402.3:c.653C>T	p.Ser218Phe	G6PD deficiency, XL

1390539	Abdominal distention, abdominal pain, abnormal biliary tract morphology, ascites, chronic pancreatitis, diarrhea, elevated hepatic transaminase, episodic vomiting, hepatomegaly, hyponatremia, pancreatic pseudocyst, recurrent pancreatitis, short stature	Hom	*AGL* NM_000028.2:c.753_756del	p.(Asp251Glufs*23)	Glycogen storage disease IIIa/IIIb, AR	Het	*CTRC* NM_007272.2:c.703G>A	p.(Val235Ile)	Chronic pancreatitis, AD/AR

Hom: homozygous, Het: heterozygous, Hem: Hemizygous, Zyg: Zygosity, Ref. Seq: reference sequence, nt: nucleotide, MOI: Mode of inheritance, AR: autosomal recessive, AD: autosomal dominant, XL: X-linked

Table 4. Research findings in cases with no clear genetic diagnosis. ***ITFG2, USP53,* and *CAP2*** were validated and confirmed as 'diagnostics' considering previous publications and our newly identified patients.

Gene	DNA change [1]	Protein change	Zyg.	Patient phenotype	Supporting evidence
CMTM6	NC_000003.11(NM_017801.2):c.138+2T>G	Predicted to affect splicing (*CMTM6* gene)	Hom	Immunodeficiency, chronic diarrhoea, splenomegaly, FTT	Role in immunity as regulator of PDL1[1]
CAPNS1	NC_000019.9(NM_001003962.1):c.781-1G>T	Predicted to affect splicing (*CAPNS1* gene)	Hom	Muscular weakness, high CPK, dystonia, peripheral axonal neuropathy	Essential for embryonic development Calpains have been implicated in neurodegenerative processes, such as myotonic dystrophy [2,3]
SLC15A3	NC_000011.9(NM_016582.2):c.1107+1G>T	Predicted to affect splicing (*SLC15A3* gene)	Hom	Immunodeficiency, chronic diarrhoea and recurrent respiratory infections, FTT	Postulated to participate in innate immune responses by regulating MAVS- and STING-mediated signalling pathways and playing an important role in regulating TLR4-mediated inflammatory responses [4,5]
CAP2	NM_006366.2:c.948T>G	p.(Tyr316*)	Hom	Atrial situs ambiguous, DCM, PDA, PFO, congestive heart failure. Two siblings deceased due to DCM	Major role in regulating the actin cytoskeleton. Knockout mice with microphthalmia and cardiac conduction disease and dilated cardiomyopathy (DCM). Hom. splicing variant detected in two children with DCM [6,7]
WNK2	NM_001282394.1:c.2736C>G	p.(Tyr912*)	Hom	Febrile seizures, hyperreflexia, hypertonia, intellectual disability, leukodystrophy, NDD, seizures. Two similarly affected siblings (hom)	Cytoplasmic serine-threonine kinase, almost exclusively expressed in the brain as well as in cortical and thalamic neurons [8]

XIRP2	NM_152381.5:c.68_69del	p.(Arg23Lysfs*2)	Hom	Anemia, chronic pancreatitis, sensorineural hearing impairment, thrombocytopenia	Xirp2-null mice revealed high frequency hearing loss and stereocilia degeneration [9]
ITFG2	NM_018463.3:c.361C>T	p.(Gln121*)	Hom	NDD, regression, seizures, ataxia. Similarly affected sibling (hom)	Harripaul *et al.*, identified a nonsense homozygous variant, c.472G>T p.(Glu158*), in members of a consanguineous family [10]
USP53	NM_019050.2: c.1524T>G	p.(Tyr508*)	Hom	Hepatomegaly, hypoalbuminemia, hypoproteinemia, intrahepatic cholestasis, jaundice, leukocytosis, prolonged prothrombin time, pruritus, thrombocytosis.	Maddirevula *et al.*, identified a homozygous truncating variant in a consanguineous family with two siblings and a cousin with a syndrome of cholestasis and hearing loss [11]
CD5L	NM_005894.2:c.631C>T	p.(Arg211*)	Hom	Elevated hepatic transaminase, hepatomegaly, hyperbilirubinemia, jaundice, pruritus.	Bárcena *et al.*, reported that this gene is a pleiotropic player in liver fibrosis controlling damage, fibrosis, and immune cell content [12]
DUSP4	NC_000008.10(NM_001394.6):c.-2118A>G	Unknown effect. *DUSP4* gene	Hom	Tetralogy of Fallot, hypertrophic cardiomyopathy. Similarly affected sibling (hom)	Dual specificity phosphatase 4 mediates cardiomyopathy caused by lamin A/C (*LMNA*) gene mutation [13]
ACSL1	NC_000004.12(NM_001286708.1):c.577+3A>C	Predicted to affect splicing *ACSL1* gene	Het	Ischemic heart disease, diabetes mellitus, elevated serum CPK, Increased lactate dehydrogenase.	ACSL1 plays a key role in both the synthesis of cellular lipids and the degradation of fatty acids. In mouse model, Zhao *et al.*, showed loss of Acsl1 in mouse skeletal muscle (Acsl1M-/-) severely

				Mother and brother similarly affected	reduces acyl-CoA synthetase activity and fatty acid oxidation [14]
SHQ1	NM_018130.2:c.850T>C	p.(Tyr284His)	Hom	Anemia, NDD, regression, dystonia, FTT, focal-onset seizures	Bizarro *et al.* identified a patient affected with a severe neurological disorder, including cerebellar degeneration with 2 heterozygous variants in this gene [15]

Hom: homozygous, Het: heterozygous, NDD: Neurodevelopmental delay, FTT: failure to thrive, DCM: dilated cardiomyopathy, PDA: patent ductus arteriosus, PFO: patent foramen ovale, CPK: creatine phosphokinase

[1] Nomenclature of DNA variants according to HGVS recommendations, including intronic variants (e.g. NC_000003.11(NM_017801.2):c.138+2T>G). All variants were checked with Mutalyzer to ensure correct nomenclature.

References

1 Mezzadra, R. *et al.* Identification of CMTM6 and CMTM4 as PD-L1 protein regulators. *Nature* **549**, 106-110, doi:10.1038/nature23669 (2017).

2 Arthur, J. S., Elce, J. S., Hegadorn, C., Williams, K. & Greer, P. A. Disruption of the murine calpain small subunit gene, Capn4: calpain is essential for embryonic development but not for cell growth and division. *Mol Cell Biol* **20**, 4474-4481, doi:10.1128/mcb.20.12.4474-4481.2000 (2000).

3 Sorimachi, H. & Ono, Y. Regulation and physiological roles of the calpain system in muscular disorders. *Cardiovasc Res* **96**, 11-22, doi:10.1093/cvr/cvs157 (2012).

4 He, L. *et al.* The Solute Carrier Transporter SLC15A3 Participates in Antiviral Innate Immune Responses against Herpes Simplex Virus-1. *J Immunol Res* **2018**, 5214187, doi:10.1155/2018/5214187 (2018).

5 Song, F. *et al.* Regulation and biological role of the peptide/histidine transporter SLC15A3 in Toll-like receptor-mediated inflammatory responses in macrophage. *Cell Death Dis* **9**, 770, doi:10.1038/s41419-018-0809-1 (2018).

6 Aspit, L. *et al.* CAP2 mutation leads to impaired actin dynamics and associates with supraventricular tachycardia and dilated cardiomyopathy. *J Med Genet* **56**, 228-235, doi:10.1136/jmedgenet-2018-105498 (2019).

7 Field, J. *et al.* CAP2 in cardiac conduction, sudden cardiac death and eye development. *Sci Rep* **5**, 17256, doi:10.1038/srep17256 (2015).

8 Rinehart, J. *et al.* WNK2 kinase is a novel regulator of essential neuronal cation-chloride cotransporters. *J Biol Chem* **286**, 30171-30180, doi:10.1074/jbc.M111.222893 (2011).

9 Francis, S. P. *et al.* A short splice form of Xin-actin binding repeat containing 2 (XIRP2) lacking the Xin repeats is required for maintenance of stereocilia morphology and hearing function. *J Neurosci* **35**, 1999-2014, doi:10.1523/JNEUROSCI.3449-14.2015 (2015).

10 Harripaul, R. *et al.* Mapping autosomal recessive intellectual disability: combined microarray and exome sequencing identifies 26 novel candidate genes in 192 consanguineous families. *Mol Psychiatry* **23**, 973-984, doi:10.1038/mp.2017.60 (2018).

11 Maddirevula, S. *et al.* Identification of novel loci for pediatric cholestatic liver disease defined by KIF12, PPM1F, USP53, LSR, and WDR83OS pathogenic variants. *Genet Med* **21**, 1164-1172, doi:10.1038/s41436-018-0288-x (2019).

12 Barcena, C. *et al.* CD5L is a pleiotropic player in liver fibrosis controlling damage, fibrosis and immune cell content. *EBioMedicine* **43**, 513-524, doi:10.1016/j.ebiom.2019.04.052 (2019).

13 Choi, J. C. *et al.* Dual specificity phosphatase 4 mediates cardiomyopathy caused by lamin A/C (LMNA) gene mutation. *J Biol Chem* **287**, 40513-40524, doi:10.1074/jbc.M112.404541 (2012).

14 Zhao, L. *et al.* Defective fatty acid oxidation in mice with muscle-specific acyl-CoA synthetase 1 deficiency increases amino acid use and impairs muscle function. *J Biol Chem* **294**, 8819-8833, doi:10.1074/jbc.RA118.006790 (2019).

15 Bizarro, J. & Meier, U. T. Inherited SHQ1 mutations impair interaction with NAP57/dyskerin, a major target in dyskeratosis congenita. *Mol Genet Genomic Med* **5**, 805-808, doi:10.1002/mgg3.314 (2017).

Information on required consent for genetic analysis

CENTOGENE requires a signed consent form from the patient in order to be legally able to conduct a genetic analysis. Please ensure that this signed consent form accompanies the sample(s).

Dear patient,

Your physician has recommended a genetic analysis for you (or a person in your legal custody) to clarify the diagnosis/symptoms stated in the section "declaration of consent" below. In order to ensure that you have understood the purpose and significance of a genetic analysis, we have provided information about the testing process and potential results below.

The purpose of a genetic analysis is to identify the cause of a suspected disease in you or your family by analyzing your genetic material (DNA) for an abnormal change (variant) that could explain the disease you or members of your family are experiencing.

In a genetic analysis, depending on the case, you can be tested for:

- A single gene/variant responsible for a specific, suspected genetic disease, or
- Multiple genes (gene panels, whole exome or genome sequencing) in parallel.

The study material that is needed to perform the genetic analysis is stated in the test order form and is typically blood or purified DNA, but may also be tissue, saliva or buccal swab.

Possible results from the genetic analysis:
A genetic analysis can have one of several outcomes:

- A disease-causing DNA variant is identified confirming the diagnosis and allowing appropriate medical management by your physician (if such is available).
- A DNA variant is identified but at this time, there is not enough scientific and medical information to determine if this is a disease-causing variant or not. Your physician will discuss such a result with you and explain what further options are available to you.
- The genetic analysis results in no specific finding that can explain the symptoms. This can be due to the current limitations in scientific or medical knowledge and technology.

It is important to understand that genetic analyses – even if the result of a specific analysis is negative - are not exhaustive and that it is therefore not possible to exclude risks for all possible genetic diseases for yourself and your family members (especially your children).

It is possible that the knowledge of the test results may result in psychological stress for you and your family. It is always recommended to discuss the results with your responsible physician.

Incidental findings:
Genetic analyses, particularly those involving a large number of genes such as whole exome or genome sequencing, may identify results that are not directly related to the actual reason for your testing (incidental findings). However, such findings could still be of medical importance for you and your family, as they may provide information about a risk (that you may not be aware of) for potentially serious, unavoidable or non-treatable genetic diseases.

As part of the optional sections of your consent declaration below, you can decide whether or not and under which circumstances you wish to be informed about such incidental findings.

Family relationship findings:
If several family members are tested, the correct interpretation of the results depends on the provided relationships between family members being accurate. If the genetic analysis reveals a possibility that there is a discrepancy in the provided relationships, CENTOGENE will not inform you, unless in exceptional cases where this information is absolutely necessary for the completion and correct medical interpretation of the requested analysis.

Use of the health data, sample and test results:
The sample and provided data including health data will be used for the requested analysis and along with the test results will be stored and processed in accordance with your consent declaration below.

Right of withdrawal:
You can withdraw your consent to the analysis with effect for the future at any time in full or in part without providing a reason.

Right not to know:
You have the right not to be informed about test results (right not to know) and to stop the testing processes that have been started at any time up to being given the results and to request the destruction of all analysis results.

Pseudonymisation and Anonymisation:

Pseudonymisation means the processing of your personal data in a way that the personal data can no longer be attributed to your person without a certain identifier, which is kept separately and protected only by CENTOGENE. “Anonymisation” refers to the process of rendering your data anonymous, which then does not allow your identification from the anonymous data at all anymore.

Data protection information for patient and physician:

In the following we want to inform you about the processing of personal data during and after the performance of the genetic analysis. "Personal data" is understood to mean all information which relates to an identified or identifiable natural person. To all such collected and processed personal data, the following applies:

- Controller and responsible entity for the processing of your personal data is CENTOGENE AG, Am Strande 7, 18055 Rostock, represented by the Executive Board members as can be found on our website (https://www.centogene.com/about-centogene/team/executive-board.html). You can reach our data protection officer under the same address with the addition “Attn: Data Protection Officer” or by email dataprivacy@centogene.com.
- Patient: By virtue of this consent form and through your physician, we collect the following data about you (in each case insofar as provided): personal details (including name and address), family relations, age/date of birth, gender, ethnicity, nationality, insurance information, symptoms and other medical information, disease, the study material / sample with identifiable genetic data, the genetic analysis results and findings. All your collected data will be stored for as long as indicated in the consent declaration. The data will be processed – partially also in data centers operated by service providers under our control and instructions - for the performance of the genetic analysis requested and for informing your physician of the results of such analysis, in each case on the basis of the consent provided. In case you have consented accordingly, such data will also be stored and processed for those further purposes as specified in the consent declaration.
- Physician: All your collected data will be processed to communicate with you about the tests and the results, as well as for invoicing, for as long as we keep identifiable data about your patients. This takes place on the basis of legal provisions allowing to process personal data for the purpose of performing a contract and for customer relation management reasons because we have a respective legitimate interest. We use data processors, which have been carefully selected and are subject to our instructions and to regular monitoring. Disclosures to data processors may result in such data being processed in countries outside of the EU (third countries). For each such transmission of data to a third country it is safeguarded that either an adequate level of protection or reasonable guarantees exist; e.g. by concluding a data processing agreement containing EU standard data protection clauses (retrievable at: https://ec.europa.eu/info/law/law-topic/ data-protection_en).
- You (Patient and Physician) do have the following rights regarding personal data relating to you, which you can exercise at any time, e.g. through an email to dataprivacy@centogene.com:

  - Right to be provided with information about and to have access to the personal data stored on you;
  - Right to have the personal data stored on you rectified or erased;
  - Right to obtain restriction of processing your personal data;
  - **Right to object on grounds relating to your particular situation;**
  - Right to data-portability (i.e. receive personal data you provided to us in a structured, commonly used and machine-readable format); and
  - Right to withdraw your consent with effect for the future at any time.

- You have the right to lodge a complaint with a supervisory authority regarding the processing of your personal data.
- You may have further or modified rights under applicable national law, which remain unaffected.
- For a more detailed and regularly updated information about how we process personal data please visit our Data Protection Statement under www.centogene.com/data-protection.

CENTOGENE
THE RARE DISEASE COMPANY

Declaration of consent

GENETIC ANALYSIS FOR DISEASE: ____________________ (filled in by the physician)

By signing this declaration of consent I acknowledge that I have received, read and understood the preceding written explanation about genetic analyses.

I also received appropriate explanations (from my physician) regarding the genetic basis, the purpose, scope, type and significance of the planned genetic analysis and achievable results, possibilities of prevention/treatment of the possible disease as well as with regard to risks associated with collecting the sample required for the genetic analysis and the knowledge of the results of the genetic analysis. All my questions have been answered and I have had the necessary time to make an informed decision about the genetic analysis.

With my signature below I give my consent or consent on behalf of the patient for whom I am the legal guardian:

(1) to the genetic analysis by CENTOGENE AG, Am Strande 7, 18055 Rostock, Germany, (CENTOGENE) for the disease stated above, (2) to the collection and processing by my physician and CENTOGENE of my "Personal (Health) Data" (meaning in particular and in each case insofar as provided: personal details (including name and address), family relations, age/date of birth, gender, ethnicity, nationality, insurance information, symptoms and other medical information, disease, the study material/sample with identifiable genetic data, the genetic analysis results and findings) as far as required to conduct the genetic analysis including any necessary transfers of my Personal (Health) Data between physician and CENTOGENE across national borders, (3) to the analysis of the obtained sample and its storage for 10 years at CENTOGENE together with my patient file to be able to verify results of the analysis if need be, (4) to add to my patient file or to files of family members and to use for the above purposes – if applicable – Personal (Health) Data on me or members of my family insofar as they have consented, (5) to inform me or my physician or – if CENTOGENE has been instructed by a laboratory acting on behalf of my physician – such laboratory about the results of the genetic analysis; and (6) to provide upon request to me, my physician or – as the case may be – the requesting laboratory, the raw data of the genetic analysis.

By ticking the relevant "YES" boxes below, I give my additional consent or consent on behalf of the patient for whom I am the legal guardian to:

Reporting of incidental findings

Whole exome sequencing (WES) and whole genome sequencing (WGS) tests analyze numerous different genes at the same time. It is therefore possible that a genetic variant found in the genetic analysis is possibly not related to the cause for ordering the testing. These findings, known as incidental findings, can provide information unrelated to your reported clinical symptoms, but can be of medical value for your treatment in the future.

I understand the significance of such incidental findings and consent to CENTOGENE reporting DNA variants of the specified classes or types in certain ☐ YES

genes in accordance with the "ACMG Recommendations for Reporting of Incidental Findings". I understand that CENTOGENE, using its own discretion, may refrain from reporting the recommended incidental findings or additionally also report (other) non-ACMG recommended incidental findings, in each case because of additional scientific and medical information available in CENTOGENE's databases.

Further storage and use of my Personal (Health) Data and the sample

I understand that my Personal (Health) Data and (remaining) sample may help in further research, development and improvement of diagnostic methods and possibly therapeutic solutions. Such measures may in the future also enable and support medical advice and guidance to me and my family members, e.g. related to the diagnosis and treatment of a potential genetic disease.

- I agree that CENTOGENE stores (1) the Personal (Health) Data I provided and information on (affected) family members - if they consented - and the results of the genetic analysis and (2) my sample (including original and processed sample) for a period of 20 years and uses this data and the remaining samples for the purpose of internal research, improvement, development and validation of analysis procedures and related product and

service developments. ☐ YES

- I agree that after a period of 20 years my Personal (Health) Data and (remaining) sample are anonymized and ownership in the sample is then transferred to CENTOGENE. Both will then remain in CENTOGENE's archives for use by CENTOGENE without restrictions.
- I agree that CENTOGENE may at any time process my anonymized or pseudonymized Personal (Health) Data, e.g. into its databases and datasets concerning genetic diseases, for the purpose of scientific and commercial research and to facilitate and contribute to the diagnosis of genetic changes and diseases of other patients. Access to such pseudonymised or anonymised data might be granted to external physicians, scientists and (pharmaceutical) companies for research and development purposes.
- I understand that I will not receive any compensation for the use of my Personal (Health) Data or sample by CENTOGENE.
- I understand that data in CENTOGENE's databases – once anonymized - cannot be destroyed upon request as it is unidentifiable and untraceable.

I am aware that I can withdraw my consent with effect for the future in full or in part at any time and that I have the right not to know the results of the genetic analyses as described in the preceding written explanation.

If the undersigning is the legal guardian of the Patient, he/she herewith to confirms to provide the above consent declarations not for himself/herself but on behalf of the respective patient.

Date	Name of Patient	Signature of Patient /Legal Guardian

I hereby confirm that the consent as shown above has been declared by the patient or (as the case may be) his/her parent or legal guardian and that I have his/her signature on file if it is not shown above. I confirm that the patient is capable of giving this consent (alternatively that the consent was given by a legal guardian of the patient), that all questions of the patient have been answered, that the patient had the necessary time to consider his/her decision and that the patient until now has not exercised his/her right not to know the results of the genetic analyses. I understand that the patient may request to have his/her genetic analyses results eliminated at any time and that I shall forward such requests to CENTOGENE without undue delay. I agree that my own personal data is stored in CENTOGENE's databases for organizational and invoicing purposes.

Date	Name of Physician	Signature of Physician
